# CAUSALRLSTACK: adaptive balancing of deep representation and causal effect estimation with application to HIV-related health data

**DOI:** 10.1186/s13040-025-00492-3

**Published:** 2025-11-05

**Authors:** Dat Thanh Pham, Khai Quang Tran, Viet Anh Nguyen

**Affiliations:** 1 Graduate University of Science and Technology, Vietnam Academy of Science and Technology, Hoang Quoc Viet, Ha Noi, 100000 Viet Nam; 2 AI VIETNAM Lab, Luong Cach, Ninh Thuan, 59000 Viet Nam; 3 Institute of Information Technology, Vietnam Academy of Science and Technology, Hoang Quoc Viet, Ha Noi, 100000 Viet Nam

**Keywords:** HIV causal estimation, Reinforcement learning, Temporal transformer, Doubly robust estimation, Ensemble method

## Abstract

**Background and Objective:**

Estimating individualized causal effects plays a vital role in data-driven decision-making, especially in high-risk domains such as public health. However, current causal inference models often lack flexibility and generalizability due to the tight coupling between representation learning and effect estimation. This study aims to develop a modular and adaptive framework to enhance the analysis of individualized causal effects in complex health data.

**Methods:**

We propose CAUSALRLSTACK, a modular framework designed to separate representation learning from causal effect estimation. In practice, the model uses a memory-augmented Transformer (TITAN) to capture complex, individualized representations. It is further paired with a doubly robust estimator(DRLearner) to improve the treatment effect estimation. A reinforcement learning agent adjusts how much each component contributes by assigning instance-specific weights. This adaptive weighting process improves the model’s ability to generalize across different populations. Input features are derived from causal graphs, automatically chosen between an expert-defined graph and one discovered from data. To evaluate performance, we applied the framework to two publicly available HIV datasets that reflect community-level testing behavior and post-intervention clinical outcomes.

**Results:**

CAUSALRLSTACK outperforms six state-of-the-art causal inference models across both datasets, achieving the highest accuracy (0.861 and 0.855), F1-Score (0.845 and 0.839), and AUC-ROC (0.897 and 0.892). It also achieves the lowest predictive uncertainty (0.093 and 0.092), indicating robust performance in estimating treatment effects.

**Conclusions:**

The proposed framework offers a flexible and effective solution for individualized causal inference. Its modular architecture and reinforcement learning-based weighting strategy enable adaptive, data-driven estimation across diverse populations. Strong experimental results demonstrate the potential of the framework to advance individualized causal inference in health data and provide a practical basis for designing personalized intervention strategies in HIV and broader public health domains.

## Introduction

Causal inference plays a central role in evidence-based decision making in applied domains such as healthcare, economics, and public policy [1, 2]. In public health, particularly in the management of the HIV epidemic, causal inference plays a critical role in guiding both preventive and treatment strategies [3, 4]. It enables researchers and policy makers to understand how specific interventions impact individual outcomes  [5–7]. Unlike traditional correlation analysis, which only describes statistical associations, causal inference seeks to uncover the underlying mechanisms that drive change [8, 9]. This distinction is critical in settings where actions must be chosen not solely based on predicted outcomes, but rather on understanding the consequences of those actions [10].

Conventional statistical methods for estimating causal effects, such as propensity score adjustment or inverse probability weighting, offer strong theoretical foundations  [8]. Various causal models are developed to address confounding factors and estimate the effects of treatment in observational health data. The inverse probability weighting with the generalized g-formula model estimates the causal effect of randomized PrEP interventions on HIV incidence using electronic health records [11]. The doubly robust causal survival model incorporates randomly assigned treatment groups to better account for confounders in the initiation of PrEP [12]. Furthermore, the causalCmprsk model [13] extends these methodologies to competing risk settings by employing inverse probability weighting within non-parametric and Cox-based frameworks to estimate average treatment effects in time-to-event data. Traditional statistical methods for causal inference have limitations, particularly in their ability to model non-linear relationships and complex interactions. These techniques often struggle with high-dimensional data.

In response, recent work has introduced neural-based causal models that integrate deep representation learning with estimation processes [14, 15]. The study referenced in  [16] employs a hybrid model that combines propensity score matching, logistic regression, and neural networks to estimate the causal impact of clinical information and prior imaging on the content of radiology reports. A deep learning model proposed using recurrent neural networks (RNN) along with disentangled representation learning is proposed in  [17] to estimate treatment effects over time from observational data, including confounders with variable time. Causal Forest  [18] utilizes decision tree ensembles to estimate heterogeneous treatment effects. Double Machine Learning (DML)  [19] merges machine learning models with orthogonal scores to reduce estimation bias. The Orthogonal Random Forest (ORF)  [20] extends DML by integrating generalized random forests for stable inference. X-Learner  [21, 22] is a meta-learning approach that is particularly effective when there is an imbalance between the treatment and control groups. The Conditional Variational Autoencoder (CEVAE)  [23] uses a variational autoencoder to model latent confounders and estimate treatment effects. Recently, the Causal Attention Transformer (CAT)  [24] was developed, which incorporates a causal understanding module into the Transformer architecture. This allows the model to learn attention weights that align with underlying causal relationships.

Despite these advances, existing models still face several limitations. Many retain fixed architectures that tightly couple representation learning with causal-effects estimation, applying the same computation pipeline to all instances. This one-size-fits-all approach limits adaptability and generalizability, especially in real-world data environments characterized by heterogeneity, distributional drift, or missing data  [7, 25]. In addition, few models explicitly support individualized decision-making through adaptive weighting or context-sensitive estimation.

Complementing these neural-based approaches, the causal inference literature has also advanced along theoretical lines, developing strategies to strengthen identifiability and estimation in observational settings, ranging from structural assumptions to representation learning frameworks. Cheng et al. [26] conducted a wide-ranging survey on data-driven methods for the estimation of causal effects in the familiar single intervention-outcome setting. Their work draws attention to key identifiability assumptions and strategies for uncovering causal structure. The study [27] addresses the fundamental challenge of hidden variables by proposing conditions that ensure identifiability and unbiased estimation. Xu et al. [28] introduce a method that combines conditional front-door adjustment with an identifiable variational autoencoder to tackle the challenge of hidden confounding in observational data. This approach strengthens identifiability and, at the same time, improves the reliability of causal effect estimates in complex settings. Louizos et al. [23] propose a disentangled representation learning framework that leverages instrumental variables to address unobserved confounding. By separating the information carried by instruments from other latent factors, their method enhances identifiability and supports more reliable estimation of causal effects. The authors in [29] introduce a disentangled representation framework for causal mediation analysis, aiming to separate direct and indirect effects through structured representations. This approach extends causal inference beyond total effects and provides a more interpretable view of mediation pathways. This study showed the potential for interpretable causal representations. However, this work and the above studies did not consider optimizing representations for adaptability under distributional shifts, which was a possible direction for improvement.

To address these challenges, we introduce CAUSALRLSTACK, a new modular framework for causal inference that separates representation learning from treatment effect estimation. It constructs two candidate causal graphs, one based on expert knowledge and another on a Rex method [30], which takes advantage of Shapley values to guide variable importance and employs a cycle removal procedure to generate a valid causal DAG. This approach follows previous recommendations on DAG validation and evaluation in applied epidemiology [31–33]. Then, sensitivity analysis is applied to select the most suitable graph as input for the modeling process.

CAUSALRLSTACK uses a memory-augmented Transformer, inspired by the TITAN architecture [34], as a representation component. With the addition of external memory, the model can store and recall contextual information across samples. This makes it more adaptable when data distributions change and more resilient to sparse or irregular patterns that are common in healthcare datasets. As a result, the framework produces more informative representations that can support causal estimation.

Individualized representations are fed into a doubly robust estimator, the DRLearner [35], which helps to ensure that the causal effect estimates remain consistent and less sensitive to bias. The DRLearner is particularly effective in this framework because it integrates smoothly with deep representations and maintains its validity even if either the outcome model or the propensity model is misspecified. This characteristic can be particularly useful in observational healthcare data, where confounders are common.

CAUSALRLSTACK incorporates a reinforcement learning (RL) agent [36] as an ensemble mechanism to combine the two components. The agent dynamically adjusts the weights for each instance. In practice, the RL-based ensemble shifts the emphasis between deep representations and statistical estimation. In some cases, it leans more on representation, while in others it gives greater weight to the estimator. This flexibility allows estimates to reflect individual differences while still accounting for bias, which may improve their relevance for causal analysis. Finally, the overall architecture incorporates predictive uncertainty estimation and distribution shift detection [7, 25], strengthening its practicality for real-world causal inference tasks.

In summary, this work makes three key contributions: (1) we propose a modular framework for causal estimation that separates representation learning from causal effect estimation; (2) we design a memory-augmented Transformer representation module, inspired by TITAN, that improves generalization under distributional shifts and handles sparse patterns in healthcare data; and (3) we introduce an RL-based ensemble mechanism that adaptively balances deep representations with statistical estimation, allowing for personalized and causally valid effect estimates.

To evaluate the performance of CAUSALRLSTACK, we used two publicly available HIV datasets covering both community-level surveys and clinical trial records. In this way, we were able to assess the robustness of the framework in diverse healthcare settings, where the experimental results indicated that CAUSALRLSTACK achieved improved performance compared to baseline methods in several evaluation metrics.

## Materials and methods

### Datasets

We used two publicly available HIV-related datasets from Kaggle for our evaluation. The first dataset is the EDHS-HIV/AIDS dataset, provided on Kaggle [37] and initially compiled from the Ethiopian Demographic and Health Survey (EDHS) conducted by the Ethiopian Central Statistical Agency (CSA) in collaboration with ICF International. This data set has been used in peer-reviewed studies [38, 39] for machine learning research. It includes 78,877 anonymized individual records and captures the behavior and risk factors of population-level HIV testing.

The second dataset is the AIDS Virus Infection Prediction dataset, provided from Kaggle [40], which is reported to be based on the AIDS Clinical Trials Group (ACTG) Study 175 [41]. The Kaggle version is a processed and expanded dataset consisting of approximately 50,000 anonymized records, released under a CC0 license. This dataset contains information related to clinical trials on HIV treatment. A more detailed description of the two datasets is provided in the following.

**1. Dataset 1 (EDHS-HIV/AIDS dataset).** Each record of dataset 1 contains structured demographic, behavioral, and HIV-related knowledge attributes. This dataset is appropriate for conducting causal inference and survival estimation analyzes in the context of HIV testing and prevention. The intervention variable is S_Test (0 = No, 1 = Yes), indicating whether a sample test was taken, and the outcome variable is T_in_LAB (0 = No, 1 = Yes), representing whether confirmatory laboratory tests occurred. The features are grouped into the following.

*Demographic and socio-economic variables:* gender, age, region, residence type, religion, education level, marital status, employment status and wealth index.

*Sexual behavior indicators:* number of sexual partners, use of condoms, and behavior changes aimed at reducing the risk of HIV.

*HIV-related knowledge:* beliefs about transmission, awareness of STIs, knowledge of HIV/AIDS, and access to testing services.

**2. Dataset 2 (AIDS Virus Infection Prediction dataset).** The second dataset focuses on clinical trials of HIV treatment. The outcome variable is infected, indicating whether a patient is infected with AIDS (1 = Yes, 0 = No). The intervention variable is trt, representing the treatment group, with four specific values: 0 = ZDV monotherapy (ZDV only), 1 = ZDV + ddI, 2 = ZDV + Zal, and 3 = ddI monotherapy (ddI only). The remaining input variables are classified into four main groups as follows.

- *Demographic and behavioral variables:* age, gender, race, drug use, and homosexual status.
- *Clinical variables:* body weight, hemoglobin, Karnofsky score, presence of symptoms, and history of opportunistic infections.
- *Immunological markers:* CD4 count, CD8 count, and CD4/CD8 ratio.
- *Treatment-related variables:* treatment type and regimen indicators.

Causal graphs constructed from these datasets, both expert-defined and data-driven, are described below.

### Data prepocessing

Our two HIV datasets contained a mix of variable types, missing values, and imbalanced treatment groups, which made a clear preprocessing strategy essential. We converted categorical variables into numeric codes by label encoding and applied robust scaling to continuous measures so that extreme values did not dominate while the results remained clinically meaningful. Approximately 8.3% of the data were missing; we addressed this by imputing continuous measures with their median values and categorical attributes with their dominant category, reflecting the distinct missingness patterns in laboratory versus survey data.

We also developed a domain-informed approach to handle missing values and a population-based relabeling method to correct treatment imbalance. Our strategy addresses imbalance while preserving genuine covariate patterns, rather than relying on interpolation methods such as SMOTE [42], which may generate patient profiles that are not clinically realistic. In this way, the preprocessing pipeline remains computationally efficient and causally reliable, providing a strong foundation for the analyses that follow.

### Problem setup and assumption

#### Problem setup

In this study, we estimate individual causal effects using observational data from real-world health-related scenarios. Our goal is to evaluate the potential impact of a specific intervention or behavioral exposure, denoted as *Intervention*, on a binary outcome, denoted as *Outcome*.

We define the input matrix $\mathbf{X} \in \mathbb{R}^{n \times p}$, where $n$ is the number of individuals and $p$ is the number of observed features. These features may include sociodemographic, behavioral, clinical, or knowledge-based variables. Among them, we focus on two main variables:

1. *Intervention* ($T$): A categorical variable representing the exposure level or the intervention status for each individual. The possible values of $T$ are denoted by the set $\mathcal{T}$, where $\mathcal{T}$ may include two or more discrete categories (e.g., $\mathcal{T} = \{0, 1, 2, 3\}$) depending on the dataset.
2. *Outcome* ($Y$): A binary variable indicating whether the individual experienced the event or outcome of interest (1 if yes, 0 if no).

Our model aims to estimate the potential outcome $Y_i(t)$ for each individual $i$, assuming they were assigned a specific intervention level $t \in \mathcal{T}$.

Specifically, the model estimates the following conditional expectation.

1 $$\mu_i(t) = \mathbb{E}\!\left[ Y_i(t) \mid X_i \right],$$

where $Y_i(t)$ denotes the potential outcome at the intervention level $t$, and $X_i$ is the vector of observed characteristics for individual $i$. We use $\mu_i(t)$ to represent the true conditional expectation, and $\hat{\mu}_i(t)$ to denote its estimator obtained by our model.

To quantify the causal effect between any two intervention levels $a, b \in \mathcal{T}$, the Individual Causal Effect (ICE) is defined as follows.

2 $$\tau_i^{(a,b)} = \hat{\mu}_i(a) - \hat{\mu}_i(b),$$

#### Identification Assumptions

To identify causal effects from observational data, we rely on the standard assumptions of the potential outcome framework.

*Consistency.* If an individual $i$ actually receives the level of treatment $t$, then the observed outcome is exactly the potential result under that treatment. In other words, $Y_i = Y_i(t)$ when $T_i = t$.

*Positivity (Overlap).* For every covariate profile $X_i$, each treatment level has a non-zero probability of being observed. Formally, $0 < P(T=t \mid X_i) < 1$ for all $t \in \mathcal{T}$.

*Ignorability (Unconfoundedness).* Conditional on the observed covariates $X_i$, the assignment of treatment is independent of potential outcomes: $Y_i(t) \perp\!\!\!\perp T_i \mid X_i$ for all $t \in \mathcal{T}$.

*SUTVA (Stable Unit Treatment Value Assumption).* There is no interference between individuals (the outcome of one person does not depend on the treatment of another person) and each treatment level is well defined.

For the first dataset (EDHS-HIV/AIDS), the treatment variable is S Test ($0 =$ No, $1 =$ Yes), and the outcome variable is T in LAB ($0 =$ No, $1 =$ Yes). In consistency, if a person actually goes for community screening, the laboratory confirmation observed is precisely the potential outcome under that condition. Ignorability assumes that once we condition on demographic, behavioral, and knowledge variables, the decision to be screened is independent of the potential laboratory confirmation. Positivity requires that in every subgroup defined by these covariates, there are screened and unscreened individuals.

For the second dataset (AIDS Virus Infection Prediction), the treatment variable is trt (treatment group: $0 =$ ZDV only, $1 =$ ZDV+ddI, $2 =$ ZDV+Zal, $3 =$ ddI only), and the outcome is infected ($0 =$ No, $1 =$ Yes). Consistency means that if a patient actually received ZDV+ddI, then the observed infection status matches the potential outcome under that regimen. Ignorability assumes that, after conditioning on demographic, behavioral, clinical, and immunological variables, the assignment of treatment is independent of potential infection outcomes. Positivity requires that, in all strata of patient characteristics, patients appear in all four treatment groups.

In practice, we checked both datasets and did not find major signs of assumption violations, so we consider them reasonable in this setting.

### Proposed methods

#### Overview of proposed framework

To address the challenges of estimating individual causal effects using observational data, we propose a comprehensive framework called CAUSALRLSTACK. This framework combines causal graph discovery with a hybrid causal estimation architecture. It has three main stages: causal graph construction, dual path modeling, and reinforcement-based adaptive integration.

In the first stage, we create two candidate causal graphs representing the potential causal relationships between input variables. One graph is constructed based on domain expert knowledge, while the other is generated directly from the data using a causal discovery pipeline, such as REX. We then analyzed the sensitivity to compare and select the optimal graph structure. This selection ensures that the model is informed by a reliable and contextually appropriate causal representation.

Based on the chosen causal graph, we extract a subset of causally relevant variables for the intervention and the outcome. This subset is represented as a feature matrix $X \in \mathbb{R}^{n \times p}$, where each row corresponds to an individual in the dataset, and each column represents an input feature retained by causal reasoning. This process eliminates irrelevant or post-intervention variables, which helps reduce bias and enhances the accuracy of estimating causal effects.

The resulting matrix $X$ is then processed by two main components as follows.

1. **TITAN-based component**, a memory-augmented Transformer-based encoder, learns deep context-sensitive representations from input features. Models non-linear interactions among these features and creates semantically rich embeddings for each instance. Additionally, its global memory and surprise state mechanisms allow for effective adaptation to unusual or rare patterns in the data.
2. **DRLearner,** a doubly robust causal estimator, combines an outcome regression model with an intervention assignment model. This approach allows for accurate estimation of both average causal effects (ACE) and individual causal effects (ICE), while remaining resilient to potential model misspecifications that can arise from noise or data limitations.

To effectively combine the outputs of both components, we utilize a reinforcement learning (RL) agent that learns a dynamic, instance-specific weighting policy. This policy optimally integrates the outputs of TITAN and DRLearner. This adaptive ensemble strategy enables the model to customize its inference for individual cases, striking a balance between rich representation and robust causal estimation.

**Fig. 1:**
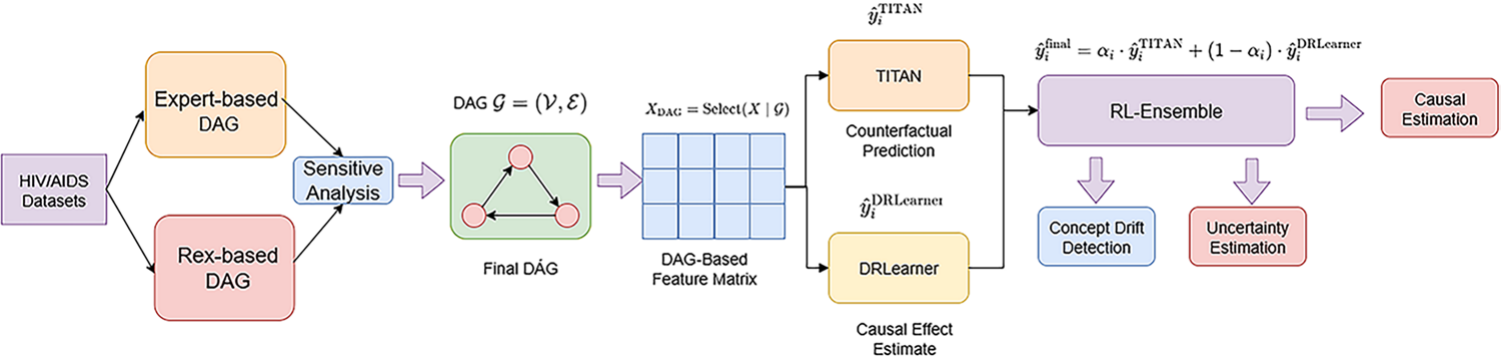
The overall architecture shows the structure of our hybrid causal prediction model. Starting from the HIV/AIDS dataset, we first build a causal graph (DAG) to select important features for causal inference. These features are then used by two models, TITAN and DRLearner, which estimate causal effects using different learning strategies. An RL-based ensemble module combines its outputs using a learned weight for each sample. This module also considers a concept drift signal to adapt to changes in data and produces an uncertainty estimate alongside the final causal prediction

By decoupling representation learning from causal effect estimation and linking them through a learnable coordination mechanism, CAUSALRLSTACK offers a flexible, accurate, and interpretable solution for individualized causal inference. The overall model architecture is illustrated in Fig. 1.

#### Causal graph construction

**1. Theoretical Formulation** The process of constructing a causal graph involves two main approaches: one that utilizes expert knowledge and the other that relies on data-driven discovery. The final structure is selected through sensitivity analysis to ensure it is suitable for downstream modeling.

**Expert-Defined Causal Graph Construction.** We define an expert-driven Directed Acyclic Graph (DAG) $\mathcal{G}_E = (\mathcal{V}, \mathcal{E}_E)$, where nodes $\mathcal{V}$ represent observed variables and edges $\mathcal{E}_E$ represent directed causal relations proposed by domain experts. The structure is constructed based on prior expert knowledge and theoretical assumptions derived from domain-specific understanding.

**Causal Graph via Data-Driven Discovery.** We develop a simplified REX framework [30] to construct a causal graph from HIV surveillance data. The pipeline includes four main steps: identifying potential parent variables using SHAP values [43] and clustering, determining edge directions using the Additive Noise Model and the Hilbert-Schmidt Independence Criterion (ANM-HSIC) method [44], removing cycles using the SHAP discrepancy, and constructing the final DAG for downstream causal inference.

*Identifying potential parent variables through SHAP and clustering.* For each target variable $X_i$, a linear regression model $\hat{f}_i$ is trained using the remaining features $X_{\setminus i}$. Shapley values $\phi_{i,j}$ are then calculated to estimate the contribution of each feature $X_j \in X_{\setminus i}$ to predicting $X_i$, defined as follows.

3 $$\phi_{i,j} = \sum_{S \subseteq F \setminus \{X_j\}} \frac{|S|!(|F| - |S| - 1)!}{|F|!} \Big[ \hat{f}_i(S \cup \{X_j\}) - \hat{f}_i(S) \Big],$$

where $F = X_{\setminus i}$ is the set of features excluding the target $X_i$, $S$ is a subset of $F \setminus \{X_j\}$, and $\hat{f}_i(S)$ is the output of the model trained on subset $S$.

To improve robustness, we applied bootstrapping and used DBSCAN to cluster features in SHAP space. The cluster with the highest mean SHAP value is selected as a candidate parent. A frequency matrix $A \in \mathbb{R}^{p \times p}$ is constructed and thresholded at $\tau$ to produce an undirected adjacency matrix as follows.

4 $$A_{ji} =\begin{cases}1, & \text{if feature } X_j\, \text{is selected as a parent of } X_i \ge \tau \\0, & \text{otherwise}\end{cases}$$

*Determining edge directions using simplified ANM-HSIC.* We perform two linear regressions for each undirected edge between a pair of variables $(X_i, X_j)$ as follows.

5 $$X_i = f(X_j) + \varepsilon \quad \text{(residual } r_{i|j}) $$

6 $$X_j = f(X_i) + \varepsilon \quad \text{(residual } r_{j|i})$$

We compare the independence of the residuals with the predictor using HSIC as follows.

7 $$\text{HSIC}(X_j, r_{i|j}) \quad \text{vs.} \quad \text{HSIC}(X_i, r_{j|i})$$

The direction with the most independent residuals is selected as the causal direction.

*Cycle removal via simplified SHAP discrepancy.* After edge orientation, the graph may contain cycles. We remove the weakest edge in each cycle using SHAP discrepancy to ensure the final structure is a DAG. Instead of using the normalized squared error formula as in the original REX, we adopt a simpler version as follows.

8 $$\delta^{(i)}_j = 1 - R^2(\phi_j, X_i)$$

where $\phi_j$ is the SHAP value of the characteristic $X_j$ when predicting $X_i$, $R^2$ is the coefficient of determination between the SHAP values and the true values of $X_i$. The edge with the highest discrepancy is removed to ensure that the DAG contains no cycles while preserving the strongest causal relationships.

*DAG construction and simplification rationale.* Unlike the original REX framework, which trains two separate models (a Deep Feedforward Network and a Gradient Boosting Trees) and merges their DAGs, we simplify the process by using only linear regression to construct a single DAG. This simplification reduces computational complexity and training costs, while also enhancing the model’s interpretability. The linear model takes the following form.

9 $$X_i = \hat{f}_i(X_{\setminus i}) = \beta_0 + \sum_{j \ne i} \beta_j X_j + \varepsilon$$

The learned coefficients $\beta_j$ reflect the influence of each predictor $X_j$ on the target $X_i$, and are used to compute the SHAP values and construct the edges of the graph.

The resulting DAG $\mathcal{G}_{\text{REX}} = (V, E)$ serves as a causal basis for subsequent tasks such as identifying confounders, selecting characteristics, and controlling for post-treatment bias. In our context, the DAG is handy for estimating the causal effect of HIV screening test behavior on laboratory test behavior. It also enables the estimation of counterfactual prediction and intervention effects with greater reliability.

*Final DAG Selection for Model Based on Sensitivity Evaluation.* In this study, we select the final DAG used for causal inference based on sensitivity analysis. Rather than relying on model loss or quantitative performance metrics, we evaluated the stability of each candidate DAG by observing changes in causal components, such as confounders and mediators, after perturbing the graph structure. Specifically, we remove the direct edge from the treatment variable to the outcome variable and analyze how the identified causal variables change. The DAG that maintains more consistent causal components is considered more robust and is selected for downstream modeling. We formalize the DAG selection process as follows:

$${G_{final}} = \arg\max_{\mathcal{G} \in \{\mathcal{G}_{EX}, \mathcal{G}_{REX}\}} \ \text{Stability}(\mathcal{G})$$

where ${G_{final}}$ denotes the final selected DAG for causal inference, $\mathcal{G}_{EX}$ represents the DAG defined by experts constructed using domain knowledge, $\mathcal{G}_{REX}$ denotes the DAG driven by data learned from observational data using the REX-based framework, and $\text{Stability}(\mathcal{G})$ is a sensitivity-based measure that reflects the robustness of the causal structure under edge perturbation, calculated based on the consistency of the causal variables identified (e.g. confounders, mediators). This approach prioritizes interpretability and structural robustness over predictive accuracy, aligning with the principles of trustworthy causal modeling.

The final DAG was selected through a multi-criteria evaluation that considered stability, parsimony, and clinical relevance. In practice, the expert-defined DAG served as an epidemiological reference during the sensitivity analysis, ensuring that the final structure remained consistent with established HIV knowledge.

**Fig. 2:**
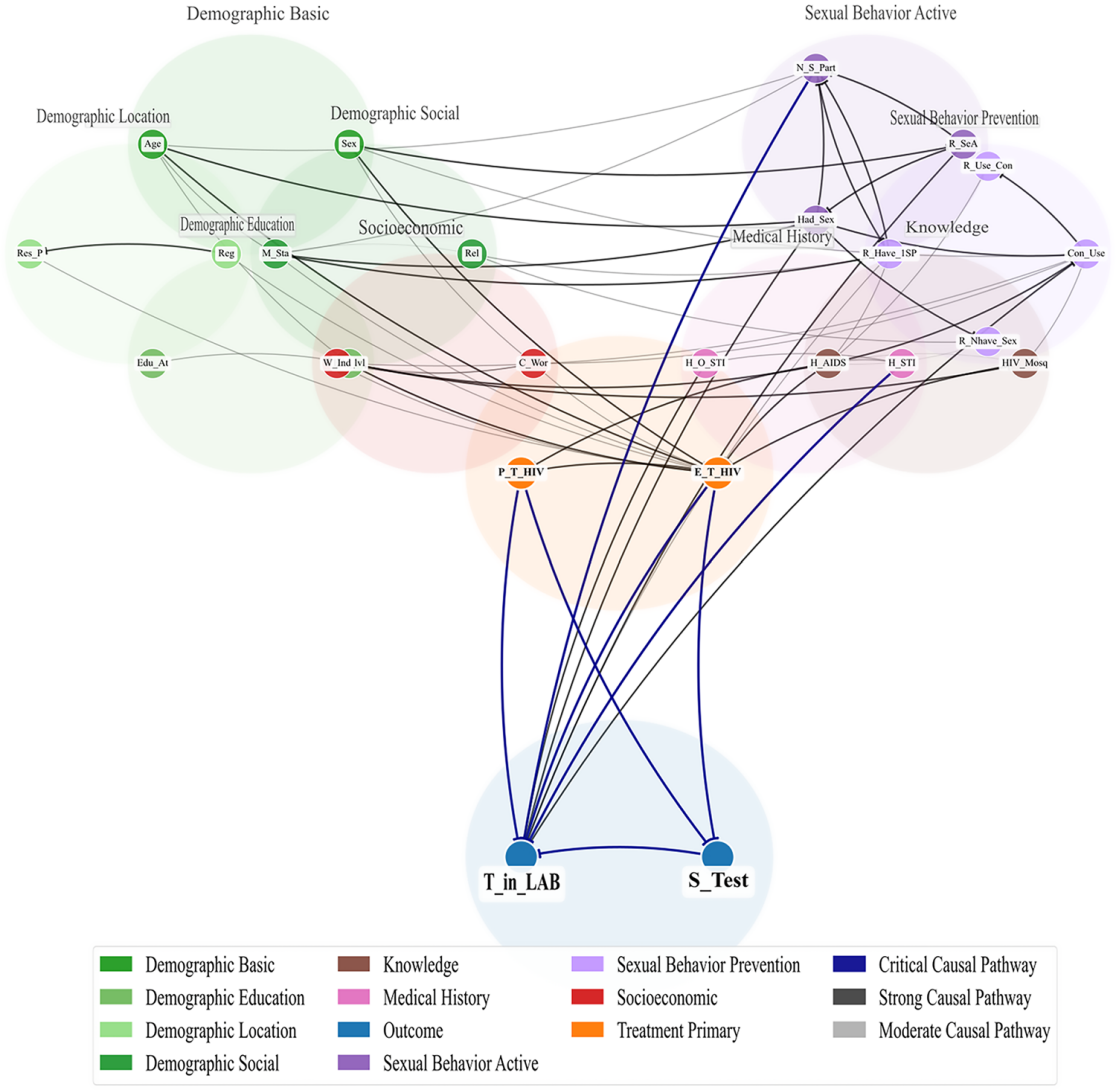
Expert-defined DAG from Dataset 1

**2.Practical Construction.** Fig. 2 illustrates the expert-defined DAG for Dataset 1. Each arrow in the graph represents an assumed causal relationship between two variables. The DAG structure reveals the following key relationships.

Sex and Age influence Reg (region) and C_Wor (current work status), which subsequently affect HIV testing behavior.

Res_P (residential place) and Edu_At (age at first education) influence Reg, indicating that living context plays an important role.

M_STI (marital status) and W_Ind (wealth index) affect both awareness and sexual behavior, such as R_SeA (availability of condoms) or Had_Sex (sexual activity).

Health-related factors like H_STI (symptoms of sexually transmitted infections) and H_AIDS (awareness of AIDS) are directly linked to decisions regarding HIV testing (P_T_HIV and E_T_HIV).

Sexual behavior factors such as Had_Sex, Con_Use (condom use), and R_Have_1SP (having one sexual partner) strongly affect whether someone has ever been tested for HIV (E_T_HIV).

E_T_HIV and P_T_HIV further influence T_HIV_LAB (HIV laboratory testing) and S_Test (successful testing).

**Fig. 3:**
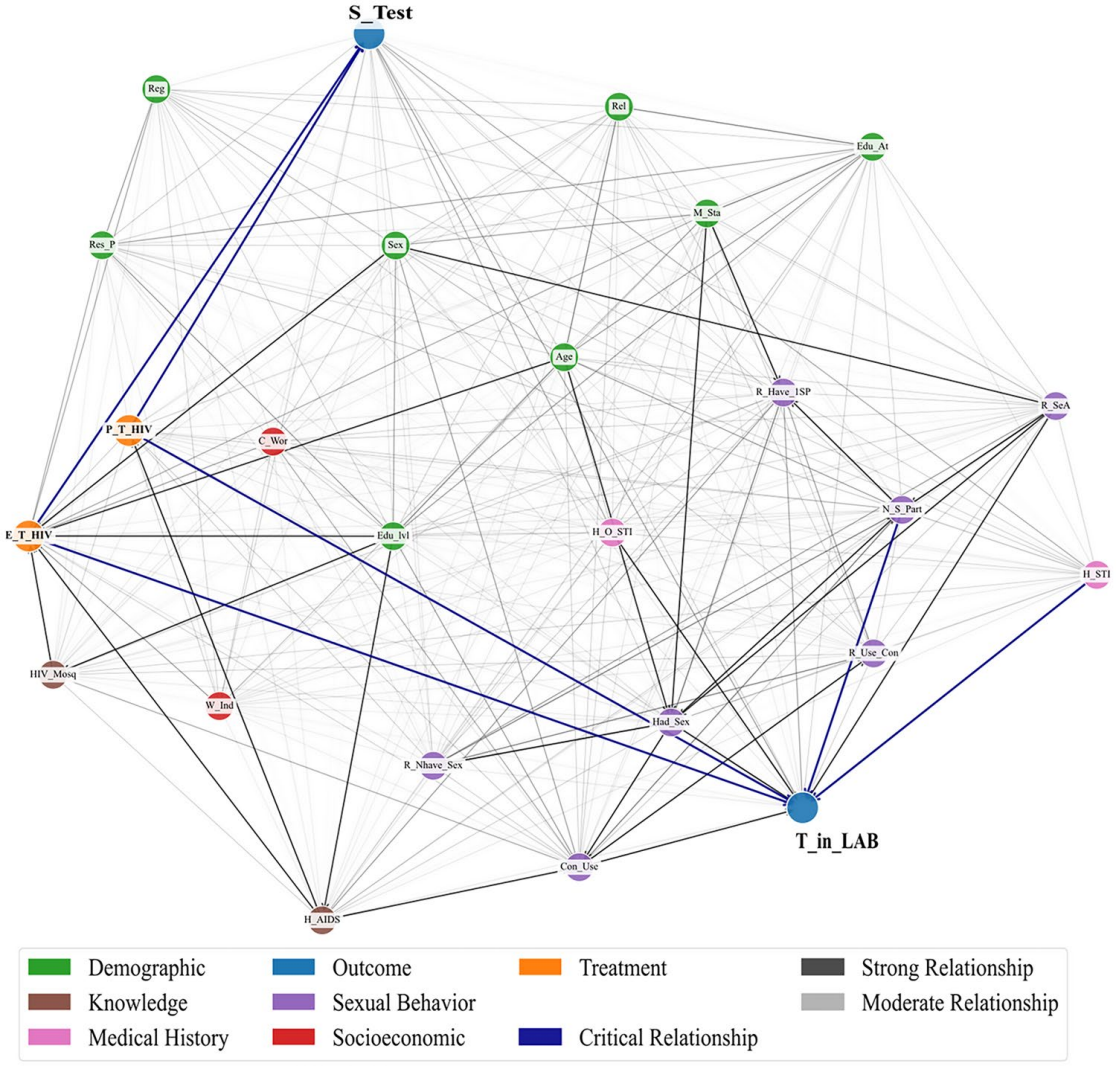
REX-inferred DAG from Dataset 1

Figure 3 presents the data-driven DAG for Dataset 1 generated using the REX causal discovery framework. Unlike the expert-defined DAG, this structure is learned directly from the data without prior domain assumptions. The graph captures a dense network of potential causal relationships between variables.

Key variables such as E_T_HIV, P_T_HIV, and S_Test are located at central positions, receiving multiple incoming and outgoing edges. Notably, several strong causal paths (in dark blue) are identified, including links from behavioral and knowledge-related factors (e.g., N_S_Part, R_SeA, Con_Use) to outcomes like HIV testing behavior (T_HIV_LAB and S_Test).

When we compared the expert-defined and data-driven DAGs for Dataset 1, we found that both graphs showed key relationships that match HIV epidemiology. Demographic factors such as sex and age influence education and work status, which then affect access to tests. The place of residence and education background also shape awareness and health-seeking behavior. Health-related factors, including a history of STIs and knowledge of AIDS, are directly related to HIV testing. The data-driven DAG suggested some additional links, such as condom use and the number of sexual partners, that make sense from a behavioral perspective. We used the expert-defined DAG as a reference in our sensitivity analysis to test the robustness of the data-driven structure. This process helped remove implausible edges and showed that the final DAG is consistent with established HIV knowledge while also adding new insights from the data.

**Fig. 4:**
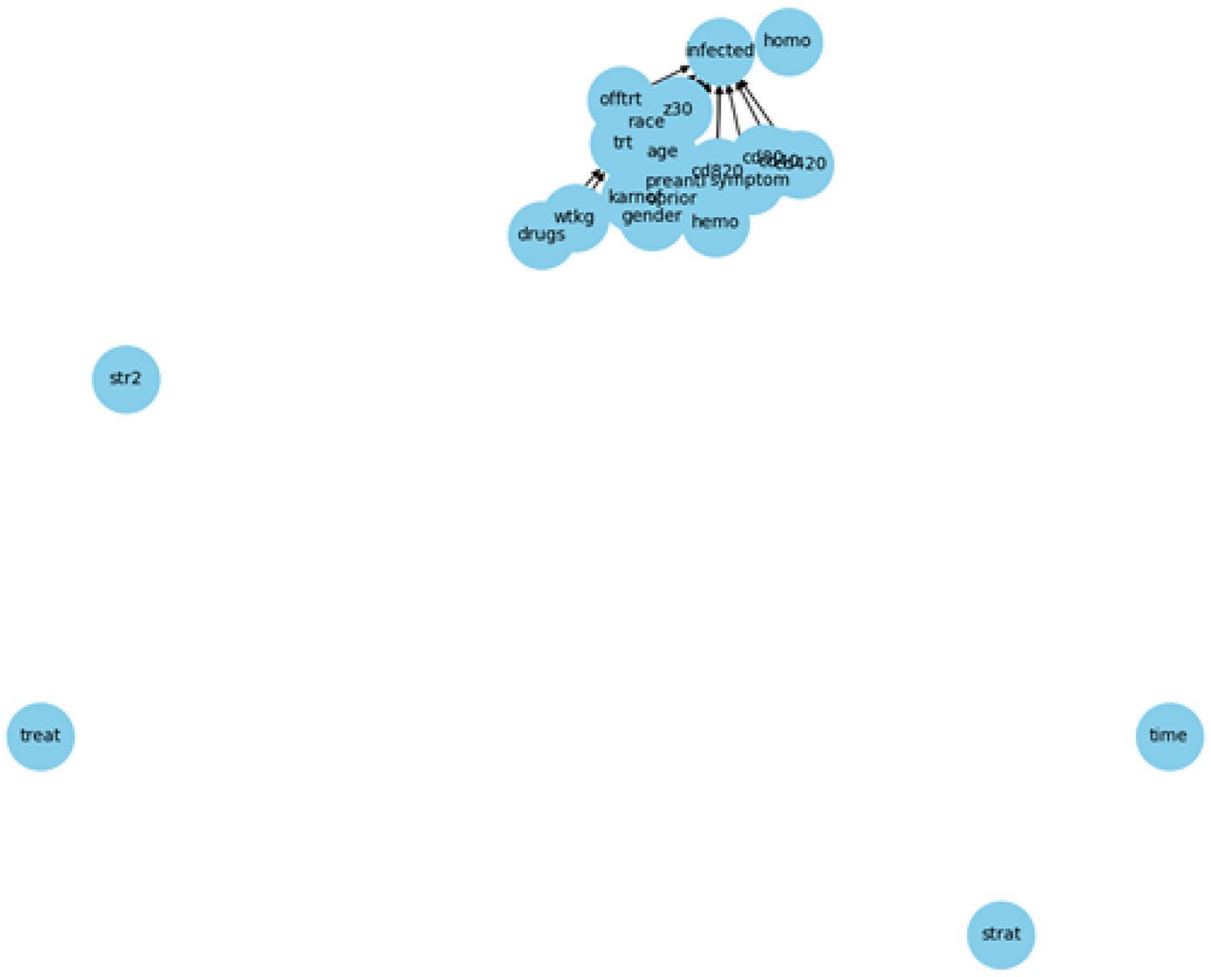
Expert-defined DAG from Dataset 2

Figure 4 illustrates the expert-defined directed acyclic graph (DAG) for Dataset 2. The graph represents hypothesized causal relationships based on knowledge of the clinical and epidemiological domain.

The variable *infected* (HIV infection status) is the central outcome, directly influenced by multiple factors including homo (homosexuality), cd420 and cd820 (immune cell markers), symtom (symptoms), z30 (reason for visit), and offtrt (treatment interruption).

Foundational characteristics such as age, gender, race, karnof (functional status), oprior (prior treatment), preanti (prior antiretroviral use), and hemo (anemia) contribute to HIV risk through intermediate clinical or immunological pathways.

Additional variables such as wtkg (weight), drugs (substance use), and trt (treatment type) are linked to background factors and can exert indirect effects on the risk of HIV infection.

Some variables such as strat, str2, time, and treat appear in the graph but do not exhibit direct causal links, likely representing stratification or temporal control variables not modeled as primary causes.

This DAG highlights the interplay between clinical characteristics, treatment history, and behavioral risk factors in shaping HIV infection outcomes. It serves as the expert-informed causal structure for Dataset 2 and is used as a candidate graph in subsequent causal analysis.

**Fig. 5:**
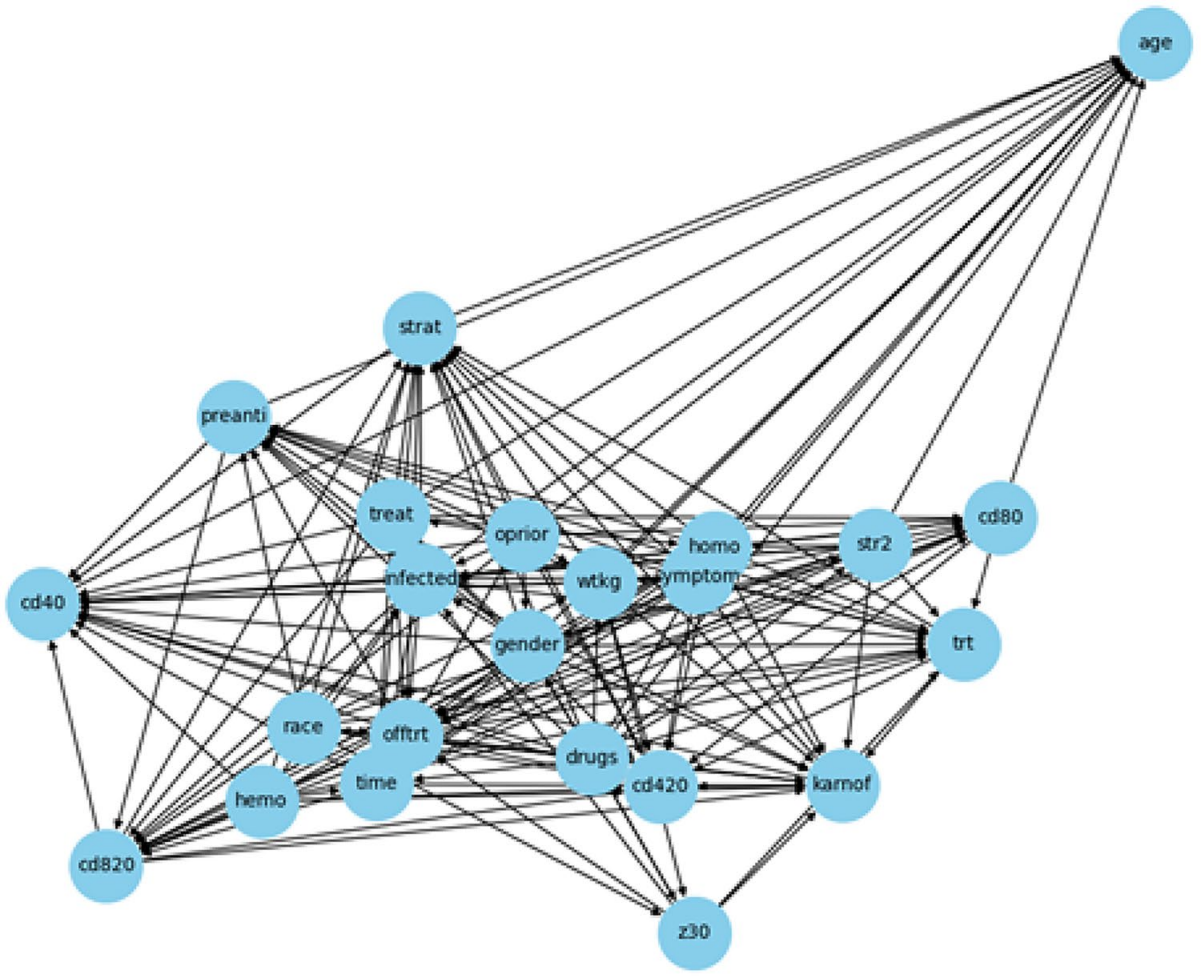
REX-inferred DAG from Dataset 2

Figure 5 illustrates the data-driven DAG inferred from Dataset 2 using the REX framework. Unlike the expert-defined DAG, this graph is constructed entirely from data, allowing the discovery of potential hidden relationships between variables.

The structure shows a high degree of connectivity among clinical, demographic, and treatment-related variables. Several variables play central roles with complex incoming and outgoing connections, reflecting the multidimensional causal dependencies present in the dataset.

Compared to the expert-defined graph, this DAG reveals a broader and more informative dependency network, supporting causal analysis and hypothesis testing based on real-world observational data.

When comparing the expert-defined and data-driven DAGs for Dataset 2, we observed that both graphs captured key relationships consistent with HIV epidemiology. For example, demographic factors such as age, sex, and race influence both risk behaviors and the likelihood of testing, while a medical history such as a prior STI is directly related to infection risk. Data-driven DAG also suggested additional associations, such as the role of treatment history (e.g. preanti, cd80), which are plausible from a clinical perspective. We used the expert-defined DAG as a reference in our sensitivity analysis to test the robustness of the data-driven structure. This procedure allowed us to filter out implausible links and demonstrate that the final DAG remains consistent with established HIV knowledge while highlighting new insights supported by the data.

*Causal Graph construction.* We construct the final DAG, denoted as$G_{final}^{dataset1}$ for Dataset 1 and $G_{final}^{dataset2}$ for Dataset 2, following the methodology described in the Proposed Method section. For each dataset, we generate two candidate DAGs: one based on expert knowledge and the other discovered using the REX causal discovery framework. We then conduct a sensitivity analysis to determine which DAG is more suitable for downstream causal modeling. The selected graph is utilized in the final model.

1. The confounders, mediators, and instrumental variables identified in $G_{final}^{dataset1}$ are as follows.

*Confounders.* A total of 11 variables: Sex, Age, Edu_lvl, Edu_At, M_Sta, C_Wor, W_Ind, H_STI, H_O_STI, H_AIDS, and Reg. These variables influence both the treatment and the outcome and must be controlled to reduce bias in causal effect estimation.

*Mediators.* A total of 4 variables: R_SeA, Had_Sex, Con_Use, and R_Have_1SP. These variables serve as causal bridges from the upstream covariates to the HIV testing behavior.

*Instrumental Variables.* Two variables: Rel and Res_P. These variables affect treatment but are not directly related to the outcome, making them useful for causal inference under potential unobserved confounding.

2. The confounders, mediators, and instrumental variables identified in $G_{final}^{dataset2}$ are as follows.

*Confounders.* A total of 11 variables: age, race, gender, karnof, oprior, preanti, cd40, cd80, wtkg, homo, and drugs. These variables influence both treatment and outcome and are controlled to reduce confounding bias in the estimation of causal effects.

*Mediators.* A total of 4 variables: cd420, cd820, symtom, and z30. These variables mediate the effect of upstream covariates on treatment behavior and are modeled as part of the causal pathway.

*Instrumental Variables.* Two variables: strat and str2. These variables are assumed to influence the assignment of treatment, but do not directly affect the outcome, supporting the identification under potential unobserved confounding.

#### TITAN for deep representation learning

Inspired by [34], we apply the TITAN (Transformer with Interpretable Temporal Attention and Neighborhood Memory) architecture to estimate the probability of an outcome in hypothetical interventions related to a binary treatment variable. TITAN is used to learn deep representations given individual covariates and treatment status. By jointly encoding demographic, behavioral, and domain-specific awareness features along with treatment assignment, TITAN captures treatment-dependent variations in a contextualized representation space.

For each individual, TITAN generates separate representations in two hypothetical treatment scenarios: $T = 1$ (treated) and $T = 0$ (not treated). TITAN can be used both independently for prediction and as a feature encoder for DRLearner, which estimates the corresponding potential outcomes $\hat{Y}(1 \mid X)$ and $\hat{Y}(0 \mid X)$.

The feature matrix $\mathbf{X} \in \mathbb{R}^{n \times p}$ is constructed based on a DAG discovered from the data. This matrix is the input of TITAN. This DAG helps identify direct causes of the target outcome and confounding variables to adjust for, and it excludes post-treatment variables to avoid bias in causal inference. This ensures that the input features reflect potential causal relationships, supporting more reliable predictions.

Each input vector $x_i \in \mathbb{R}^d$ representing the characteristics of an individual is passed through a one-dimensional convolutional layer (1D convolution) to capture local interactions between related feature groups as follows:

10 $$ z_i^{(0)} = \text{Conv1D}(x_i)$$

The model then employs multi-head self-attention [45] layers to learn long-range dependencies between features, allowing it to focus on the most influential factors related to the outcome. The attention mechanism is defined as:

11 $$\text{Attention}(Q, K, V) = \text{softmax}\left(\frac{QK^\top}{\sqrt{d_k}}\right)V$$

where $Q$ (Query), $K$ (Key), and $V$ (Value) are matrices derived from linear projections of the input representations, and $d_k$ is the dimension of the key vectors used for normalization to stabilize gradient updates during training. This formulation enables the model to weigh the relevance of each feature, allowing for deeper contextual representations.

The attention mechanism operates in a way that mirrors how domain experts prioritize specific risk factors when evaluating the likelihood of an outcome. For example, when evaluating an individual with several contributing factors, the model can focus more on the most critical variables while giving less weight to those that provide less information. This dynamic weighting enables the model to create more nuanced contextual representations tailored to each individual’s unique risk profile. The feature representation is then updated through the following layers.

12 $$z_i^{(\ell)} = \text{LayerNorm}\left(z_i^{(\ell-1)} + \text{MultiHeadAttention}(z_i^{(\ell-1)})\right)$$

13 $$z_i^{(\ell)} = \text{LayerNorm}\left(z_i^{(\ell)} + \text{FeedForward}(z_i^{(\ell)})\right)$$

Next, to improve generalization and support learning from less frequent patterns, TITAN incorporates an external memory $M \in \mathbb{R}^{m \times d}$ to store latent representations of previously observed individuals. The memory update is controlled by a sigmoid gate as follows.

14 $$g_i = \sigma(W_g z_i^{(L)} + b_g)$$

15 $$M_i = g_i \odot M_{i-1} + (1 - g_i) \odot z_i^{(L)}$$

where $g_i$ controls the level of inheritance from previous memory and $z_i^{(L)}$ is the representation of the last attention layer.

The final prediction is made by concatenating the representations based on attention and memory as follows:

16 $$\hat{y}_i = \sigma(W_o [z_i^{(L)} \, \| \, M_i] + b_o)$$

TITAN is trained using binary cross-entropy loss [46] to maximize the likelihood of accurately predicting the observed binary outcome as follows:

17 $$\mathcal{L}_{\text{TITAN}} = -\frac{1}{n} \sum_{i=1}^{n} \left[y_i \log \hat{y}_i + (1 - y_i) \log (1 - \hat{y}_i)\right]$$

where $y_i \in \{0, 1\}$ is the true observed outcome and $\hat{y}_i \in (0, 1)$ is the predicted probability.

TITAN learns contextualized non-linear representations from data through the attention mechanism, which highlights the most influential features, and through the memory component, which leverages information from similar individuals. In this study, we extend the role of TITAN beyond mere representation learning by training the model directly to estimate probabilities via the cross-entropy loss. TITAN jointly learns the representations of covariates and outcome risks in hypothetical interventions. Furthermore, we simplify the original TITAN model to increase its feasibility in real-world datasets. Instead of using complex gradient-based and surprise-based memory updates, we use a linearly updated neighborhood memory. This design preserves the model’s ability to generalize from historical data while ensuring efficiency and ease of implementation in practical settings.

#### DRLearner for causal effect estimation

Our prediction architecture incorporates DRLearner [35] as a parallel branch. DRLearner is a causal inference model capable of estimating the effect of having been tested for HIV on the risk of HIV infection. It belongs to the class of doubly robust methods which combines two complementary models: an outcome regression model and a propensity score model. This combination enables DRLearner to mitigate estimation bias even when only one of the two models is correctly specified.

Similarly to the TITAN model, the input feature matrix $\mathbf{X} \in \mathbb{R}^{n \times p}$ is derived from a DAG to ensure causal validity. For each individual $i$, DRLearner first trains two separate outcome regression models to estimate the potential outcomes under the treatment and control conditions as follows.

18 $$ \mu_1(x_i) = \mathbb{E}[Y_i \mid X_i = x_i, T_i = 1], \quad \mu_0(x_i) = \mathbb{E}[Y_i \mid X_i = x_i, T_i = 0],$$

where $Y_i \in \{0, 1\}$ represents the observed HIV infection status, $T_i \in \{0, 1\}$ denotes the treatment variable indicating whether the individual has ever been tested for HIV, and $x_i \in \mathbb{R}^p$ is the feature vector of individual $i$. Here, $\mu_1(x_i)$ and $\mu_0(x_i)$ denote the true conditional expectations of the potential outcomes under treatment and control, respectively. Their estimators obtained by DRLearner are denoted by $\hat{\mu}_1(x_i)$ and $\hat{\mu}_0(x_i)$.

Next, a propensity score model $\hat{e}(x_i) = \mathbb{P}(T_i = 1 \mid X_i = x_i)$ is trained to estimate the probability that individual $i$ was tested for HIV. This model is used to adjust for sample selection bias and is typically implemented via logistic regression or probabilistic classifiers.

DRLearner then combines the outcome and propensity models using the doubly robust correction formula as follows.

19 $$ \hat{\tau}_i = \left( \frac{t_i - \hat{e}(x_i)}{\hat{e}(x_i)(1 - \hat{e}(x_i))} \right) \cdot \left( y_i - \hat{\mu}_{t_i}(x_i) \right) + \hat{\mu}_1(x_i) - \hat{\mu}_0(x_i)$$

where $\hat{\tau}_i$ denotes the individual intervention effect (ITE) [47] for sample $i$, reflecting the change in infection probability if the individual had been tested instead of not tested, after correcting for confounding through both models.

In practical terms, this formula provides a safeguard against model misspecification. Even if the outcome or propensity score model is incorrectly specified, the doubly robust property ensures that consistent estimates of causal effects can still be obtained. This is particularly valuable in real-world applications where a single modeling approach may not fully capture the complex relationships between interventions and results.

Finally, the average intervention effect (ATE) [1] is calculated as follows.

20 $$ \mathrm{ATE} = \frac{1}{n} \sum_{i=1}^{n} \hat{\tau}_i$$

ATE quantifies the average effect of the intervention on the outcome in the population. DRLearner functions as an independent causal estimation module. Provides stable and interpretable output. DRLearner is effective in contexts that require clear causal structures and high interpretability.

#### Implementation details of DRLearner

We provide here the practical specifications of the doubly robust learner, covering outcome regressions, propensity score estimation, and the extension to multi-arm treatments.

*Outcome Regression Models.* We employ RandomForestRegressor with the following hyperparameters: (i) n_estimators = 100, validated through cross-validation; (ii) max_depth = 5, to prevent overfitting while capturing non-linear interactions; (iii) min_samples_leaf = 50, ensuring sufficient sample sizes in each terminal node; (iv) bootstrap sampling enabled, providing variance estimation and improved robustness.

*Propensity Score Models.* For treatment assignment modeling, we use LogisticRegression with L2 regularization. The regularization strength is set as $C = 1.0$, optimized via grid search. We allow up to 1000 iterations with a convergence tolerance of $10^{-4}$, and use the liblinear solver, which is robust for moderate sample sizes.

*Multi-arm Treatment Handling.* For Dataset 2 containing four treatment arms ($T \in \{0,1,2,3\}$), we extend the doubly robust estimator to handle multiple treatment contrasts. The causal effect between treatment $a$ and $b$ for instance $i$ is given by follows.

21 $$\hat{\tau}_{i}(a,b) =\frac{\mathbf{1}[T_i = a]}{\hat{e}_a(X_i)} \left(Y_i - \hat{\mu}_a(X_i)\right)- \frac{\mathbf{1}[T_i = b]}{\hat{e}_b(X_i)} \left(Y_i - \hat{\mu}_b(X_i)\right)+ \hat{\mu}_a(X_i) - \hat{\mu}_b(X_i),$$

where $\hat{e}_t(X_i) = P(T_i = t \mid X_i)$ denotes the generalized propensity score and $\hat{\mu}_t(X_i) = \mathbb{E}[Y_i \mid T_i = t, X_i]$ is the corresponding outcome regression.

This extension allows the framework to accommodate multi-arm clinical treatments, addressing the reviewer’s concern that the methodology could be restricted to binary treatment settings.

To reduce overfitting and ensure out-of-sample validity of counterfactual predictions, we applied a 5-fold cross-validation scheme. Outcome and propensity models are trained in four folds and tested on the held-out fold. This procedure also allows us to monitor the balance of estimated propensity scores across folds.

#### RL-based ensemble strategy for individualized causal estimation

Selecting the most appropriate reasoning path for each individual is crucial to enhance the accuracy of the predictions and the interpretability of the model in a complex causal estimation framework. However, traditional ensemble methods often lack the flexibility to accommodate the diversity of input features and the varying confidence levels of base models. To address this limitation, we use reinforcement learning (RL) [48] as a dynamic coordination mechanism. This approach enables the model to learn specific blending strategies tailored to individual cases, ultimately improving counterfactual predictions.

RL is a sequential decision making method in which an agent learns from interactions to maximize cumulative rewards. In this study, we adopt the RL-based Ensemble method in [36] to learn an optimal blending mechanism between TITAN and DRLearner. Instead of relying on static ensemble strategies such as averaging or fixed-weight voting, we design a meta-controller using reinforcement learning to determine the best instance-specific blending weight. This mechanism enables the model to select the most suitable causal reasoning pathway based on the input features.

Specifically, we formulate the blending task as a Markov Decision Process (MDP) [49], defined by a 5-tuple $(S, A, P, R, \gamma)$. At time $t$, the state $s_t \in S$ includes the predictions of TITAN and DRLearner, along with auxiliary characteristics such as confidence scores and bias indicators. The action $a_t \in A$ represents a blend weight $\alpha_t \in [0, 1]$, indicating the relative emphasis on each model. The reward $r_t \in R$ reflects the accuracy of the final prediction. The transition function $P$ models the probability of moving to the next state $s_{t+1}$, and $\gamma \in (0,1)$ is the discount factor for future rewards.

We use an actor-critic architecture [50] to learn the blending policy. The actor-network receives the current state $s_t$ and outputs the action $a_t = \alpha_t$, the weight of the blend between TITAN and DRLearner. The critic network estimates the action value function $Q(s_t, a_t)$, which evaluates the quality of the selected action. The actor’s parameters are updated using the policy gradient formula as follows.

22 $$\theta \leftarrow \theta + \alpha \nabla_\theta \log \pi_\theta(a_t \mid s_t) Q(s_t, a_t)$$

Here, $\theta$ denotes the parameters of the actor network, $\alpha$ is the learning rate, $\pi_\theta(a_t \mid s_t)$ is the probability of selecting the action $a_t$ in state $s_t$, and $Q(s_t, a_t)$ is the estimated action value of the critic.

The final prediction is computed as a convex combination of the two model outputs as follows.

23 $$\hat{y}_i = \alpha_i \cdot \hat{y}_i^{\text{TITAN}} + (1 - \alpha_i) \cdot \hat{y}_i^{\text{DRLearner}}$$

where $\hat{y}_i$ is the counterfactual probability of HIV infection, for example $i$, $\hat{y}_i^{\text{TITAN}}$ and $\hat{y}_i^{\text{DRLearner}}$ are predictions from the respective models, and $\alpha_i \in [0, 1]$ is the learned blending weight.

The reward $r_i$ is defined as the binary negative cross-entropy loss between the true label $y_i$ and the final prediction $\hat{y}_i$:

24 $$r_i = -\mathcal{L}_{\text{BCE}}(\hat{y}_i, y_i) = -\left[ y_i \log \hat{y}_i + (1 - y_i)\log(1 - \hat{y}_i) \right]$$

Here, $\mathcal{L}_{\text{BCE}}$ denotes the binary cross-entropy loss. This reward formulation encourages the RL agent to learn a blending policy that maximizes predictive accuracy.

To ensure stable and efficient training, we integrate three well-established reinforcement learning techniques as follows.

1. We apply the experience replay [51]. The agent stores the transitions $(s_t, a_t, r_t, s_{t+1})$ in a replay buffer $\mathcal{D}$. During training, mini-batches are sampled randomly from $\mathcal{D}$ to break temporal correlations and enhance convergence. These samples are used to update both the actor and critic via stochastic gradient descent.

2. We inject Ornstein-Uhlenbeck noise [52] to encourage exploration in continuous action spaces. The noise process is defined as follows.

25 $$\mathcal{N}_{t+1} = \mathcal{N}_t + \theta(\mu - \mathcal{N}_t)\Delta t + \sigma \sqrt{\Delta t} \cdot \epsilon_t$$

where $\mathcal{N}_t$ is the current noise value, $\theta$ is the reversion speed, $\mu$ is the long-term mean, $\sigma$ is the volatility and $\epsilon_t \sim \mathcal{N}(0,1)$ is Gaussian noise. OU noise produces smooth exploration behavior compared to uncorrelated noise.

3. We employ soft target updates using Polyak averaging [53] to update the target critic network as follows.

26 $$\theta^{\text{target}} \leftarrow \tau \theta + (1 - \tau)\theta^{\text{target}}$$

Here, $\theta$ and $\theta^{\text{target}}$ are the current and target network parameters, and $\tau \in (0,1)$ (typically $\tau = 0.001$) controls the smoothness of the update.

These techniques allow the RL agent to learn a stable and instance-specific blending strategy. This enables accurate counterfactual reasoning across groups of individuals who differ in their features.

#### Causal-aware reward and state design

To ensure unbiased causal estimation, we extend the reward function and state-space design to directly incorporate causal signals.

*Reward Function.* The primary component is a doubly robust loss, which directly measures the accuracy of causal effect estimation as follows.

27 $$r_{\text{DR}} = - \left( \frac{T_i - \hat{e}(X_i)}{\hat{e}(X_i)(1-\hat{e}(X_i))} \cdot (Y_i - \hat{\mu}_{T_i}(X_i)) + \hat{\mu}_1(X_i) - \hat{\mu}_0(X_i) - \hat{\tau}_{\text{true}}\right)^2$$

Here, $\hat{e}(X_i)$ denotes the estimated propensity score, $\hat{\mu}_t(X_i)$ the outcome regression under treatment $t$, and $\hat{\tau}_{\text{true}}$ the ground-truth effect in simulations.

In addition to this core component, we introduce: (i) *Uncertainty-weighted rewards*, which encourage the agent to down-weight uncertain predictions using confidence intervals and bootstrap estimates; (ii) *Balance-aware penalties*, which discourage covariate imbalance between treated and control groups as follows.

28 $$r_{\text{balance}} = - \sum_k \left\| \bar{X}_{k,T=1} - \bar{X}_{k,T=0} \right\|^2$$

where $\bar{X}_{k,t}$ is the weighted mean of covariate $k$ under treatment $t$ according to the implied weights of the ensemble.

*State Space.* The state representation is extended to embed causal information as follows.

29 $$\begin{aligned}s_t = [&\hat{y}^{\text{TITAN}},\; \hat{y}^{\text{DRLearner}},\; u^{\text{TITAN}},\; u^{\text{DRLearner}}, \\&\hat{e}(X_i),\; \text{overlap indicator},\;\text{covariate balance score}]\end{aligned}$$

where $u$ denotes the uncertainty of the prediction. This ensures that the RL agent’s decisions are guided by causal validity indicators rather than predictive accuracy alone. Overall, this causal-aware design directs the ensemble toward weighting strategies that enhance counterfactual reasoning and improve causal estimation quality.

#### Uncertainty estimation

We incorporate an uncertainty estimation mechanism to assess the reliability of counterfactual predictions and estimated causal effects. This enables the model to distinguish between cases with clear causal links and those with higher ambiguity. As a result, the trustworthiness and interpretability of the model improve in evaluating the causal impact of HIV screening test behavior on laboratory test behavior. In causal inference tasks using observational data, evaluating the counterfactual outcome and the reliability of such estimates is crucial. To quantify uncertainty in counterfactual predictions and the estimation of causal effects, we apply the Monte Carlo Dropout (MC Dropout) [54] method during inference.

Specifically, the model performs $T$ stochastic forward passes with dropout enabled, producing a set of counterfactual predictions $\{\hat{y}_1, \hat{y}_2, \ldots, \hat{y}_T \}$. Each $\hat{y}_t$ represents the estimated probability of HIV infection under counterfactual conditions. The predictive mean is calculated as follows.

30 $$\bar{y} = \frac{1}{T} \sum_{t=1}^{T} \hat{y}_t$$

The general predictive uncertainty is measured as the variance of the predictions as follows.

31 $$\text{Var}(y) = \frac{1}{T} \sum_{t=1}^{T} \hat{y}_t^2 - \left( \frac{1}{T} \sum_{t=1}^{T} \hat{y}_t \right)^2$$

If the model is also trained to output the aleatoric variance $\sigma_t^2$ for each forward pass, the total predictive uncertainty can be decomposed into two components as follows.

1. Aleatoric uncertainty [55] captures irreducible variability in potential outcomes due to inherent randomness or noise in the data, even under a well-specified causal model.
  32 $$\text{Aleatoric} = \frac{1}{T} \sum_{t=1}^{T} \sigma_t^2$$

2. Epistemic uncertainty [55] arises from limited knowledge about the true data-generating process and can be reduced by observing more data or improving the model.
  33 $$\text{Epistemic} = \frac{1}{T} \sum_{t=1}^{T} \mu_t^2 - \left( \frac{1}{T} \sum_{t=1}^{T} \mu_t \right)^2$$

where $\mu_t$ denotes the predicted mean in the $t$-th forward pass. Overall, the total predictive variance is represented as follows.

34 $$\text{Var}(y) = \text{Epistemic} + \text{Aleatoric}$$

This decomposition enables the model to assess the confidence of each counterfactual prediction, thereby improving interpretability and trustworthiness in public health applications such as evaluating the causal impact of the HIV screening test behavior on laboratory tests behavior.

#### Concept drift detection

The proposed model incorporates a mechanism to detect concept drift. This mechanism identifies shifts in the distribution of input features between the treatment and control groups. If these shifts are not addressed appropriately, they can reduce the predictive accuracy of the outcome and propensity models. When drift is detected, the system adjusts the ensemble weights and may also modify the causal inference method. This enhances the model’s flexibility and stability. Drift detection serves as a quality check at the input level. It enables the model to manage data inconsistencies between groups, which often occur in real-world scenarios.

We incorporate a concept drift detection mechanism [56] into the model to enhance its robustness. This component checks whether the input distribution has changed compared to training. It works by comparing the mean vector of the current input data $\mu_{\text{test}}$ with the training distribution $\mu_{\text{train}}$. A drift is flagged when the difference exceeds a defined threshold as follows.

35 $$\left\| \mu_{\text{train}} - \mu_{\text{test}} \right\| > \delta$$

This simple condition helps the model recognize significant distribution shifts. Once drift is detected, the model may adjust its ensembling weights or trigger an update. This adaptation ensures robustness in real-world deployment. It is advantageous for causal effect estimation tasks, where behavioral trends can change over time.

## Exprimental results

We first evaluate the framework on simulated data with known ground-truth causal effects, which enables us to quantify estimation accuracy under controlled conditions and validate causal correctness. We then apply the framework to real-world HIV datasets, where the goal is to assess its practical.

### Evaluation metrics

We use a common set of metrics to evaluate the framework in both simulated and real-world HIV datasets. The goal is to measure not only the predictive accuracy but also the quality of causal estimation and the reliability of uncertainty estimates.

For causal validity, we report several metrics that quantify the accuracy of treatment effect estimation. The precision in the estimation of heterogeneous effect (PEHE) is defined as

$$\text{PEHE} = \sqrt{\frac{1}{n}\sum_{i=1}^n (\hat{\tau}_i - \tau_i)^2},$$

which evaluates the root mean squared error between estimated and true individual treatment effects (ITEs). The error and bias of the Average Treatment Effect (ATE) are measured as

$$\text{ATE Error} = \big|\widehat{\text{ATE}} - \text{ATE}\big|, \quad \text{ATE Bias} = \widehat{\text{ATE}} - \text{ATE}.$$

We also compute the coefficient of determination for ITEs,

$$R^2 = 1 - \frac{\sum_{i=1}^n (\hat{\tau}_i - \tau_i)^2}{\sum_{i=1}^n (\tau_i - \bar{\tau})^2},$$

and the mean absolute error of ITEs,

$$\text{ITE MAE} = \frac{1}{n}\sum_{i=1}^n \big|\hat{\tau}_i - \tau_i\big|.$$

The estimated Average Treatment Effect (Estimated ATE), defined as the sample mean of the predicted individual treatment effects as follows.

$$\widehat{\text{ATE}} = \frac{1}{n}\sum_{i=1}^n \hat{\tau}_i.$$

This provides a direct estimate of the population-level treatment effect from the model. These measures capture how close the model is to the true causal effects at both the individual and population levels.

To examine distributional reliability, we report the standard deviation of estimated ITEs (ITE Std), the coverage rate of the true effect within the 95% confidence intervals, and the calibration error. Coverage is defined as follows.

$$\text{Coverage} = \frac{1}{n}\sum_{i=1}^n \mathbf{1}\{\tau_i \in \widehat{CI}_i^{95\%}\},$$

which measures the proportion of true effects that fall within the predicted confidence intervals. A well-calibrated model should achieve coverage close to the nominal 95%. Calibration error is quantified as the deviation between the empirical coverage and the nominal confidence level. These uncertainty metrics are only available in the simulation study, since the ground-truth causal effects are known there but not in real-world data.

For predictive performance, we use standard classification metrics: Accuracy, F1-Score, and AUC-ROC. These are consistently reported for both the simulation and HIV datasets to give a comparable view of how well the framework discriminates the outcomes. WThese metrics capture different aspects of model quality and are applicable regardless of whether the prediction target is testing behavior or cinical trial.

*1. Accuracy* measures the overall proportion of correct predictions, including positive and negative outcomes. It is calculated as follows.

$$ \text{Accuracy} = \frac{TP + TN}{TP + TN + FP + FN}$$

where *TP* (true positives) and *TN* (true negatives) refer to correctly predicted outcomes, while *FP* (false positives) and *FN* (false negatives) represent incorrect predictions. This metric provides a general overview of the predictive reliability of the model to identify positive and negative outcomes.

*2. F1-score* balances precision and recall, offering a robust performance measure in the presence of class imbalance as follows.

$$ \text{F1} = 2 \cdot \frac{\text{Precision} \cdot \text{Recall}}{\text{Precision} + \text{Recall}}, \quad \text{Precision} = \frac{TP}{TP + FP}, \quad \text{Recall} = \frac{TP}{TP + FN}$$

This metric is particularly important in settings where the consequences of misclassification can lead to substantial negative outcomes.

*3.ROC-AUC* (Receiver Operating Characteristic – Area Under the Curve) quantifies the ability of the model to distinguish between classes across all possible decision thresholds:

$$ \text{AUC} = \int_{0}^{1} TPR(FPR) \, dFPR$$

where *TPR* (true positive rate) and *FPR* (false positive rate) describe the sensitivity and fallout of the model at varying thresholds. ROC-AUC captures the model’s ability to distinguish between classes across various decision thresholds, making it especially useful in applications where classification criteria may vary.

### Simulation study

We first evaluate the framework on simulated data with known ground-truth causal effects, which allows us to assess estimation accuracy under controlled conditions. This step provides a validation of causal correctness before applying the method to HIV datasets.

#### Data generating process

We simulate a semi-realistic HIV causal system with measured confounders, a policy-like instrumental variable, a mediator, and age-modulated heterogeneous treatment effects. Unless otherwise stated, we generate $n=5000$ samples with random seed $=42$ for reproducibility. For each unit, exogenous covariates $X_{0:9}$ are drawn as follows. $X_0 \sim \mathcal{N}(50,10^2)$, $X_1 \sim \mathrm{Bern}(0.5)$, $X_2, X_3, X_9 \sim \mathcal{N}(0,1)$, $X_4 \sim \mathrm{Bern}(0.2)$, $X_5 \sim \mathrm{Unif}(0,1)$, $X_6 \sim \mathrm{Poisson}(2)$, $X_8 \sim \mathrm{Bern}(0.6)$. A latent health factor $X_7 \sim \mathcal{N}(0,1)$ is **unobserved** in the analysis dataset. An instrumental variable $Z$ depends on the geography as follows.

$$\Pr(Z=1 \mid X) = \sigma(0.8X_8 - 0.5),$$

and has no direct path to the outcome.

The assignment of treatment follows a logistic model with measured confounding and the instrument as follows.

$$\text{logit }\Pr(T=1 \mid X,Z) = 0.02(X_0 - 50) + 0.6X_2 + 0.5X_4 + 1.5Z + \varepsilon_T,$$

yields the propensity score used to draw $T$.

A post-treatment mediator $M$ (viral load-like) is generated as follows.

$$M = 2 - 1.5T + 0.5X_3 + \varepsilon_M,$$

so treatment reduces $M$ on average. The individual treatment effect is modeled as a function of age, as specified below.

$$\tau_i = -1.5 + 0.08(X_{0i} - 50).$$

The binary result is generated according to the following logistic model.

$$\text{logit }\Pr(Y=1) = 0.03(X_0-50) - 0.8X_2 + 0.7X_4 + 0.5M + 0.4X_7 + T \cdot \tau + \varepsilon_Y.$$

We release two tables per simulation: (i) the *observed* dataset $D_{\text{obs}} = \{X_0,X_1,X_2,X_3,X_4,X_5,X_6,X_8,X_9,Z,M,T,Y\}$, where $X_7$ is omitted, and (ii) a table *ground-truth* with the true ITE $\tau$, the propensity score and the $Y$-probabilities for diagnostics.

The design induces non-trivial confounding, mediation, and age-modulated heterogeneity while preserving overlap through a smooth propensity, enabling unbiased benchmarking of ATE/ITE metrics against known truth.

#### Causal graphs: ground Truth vs. Estimated

**Fig. 6:**
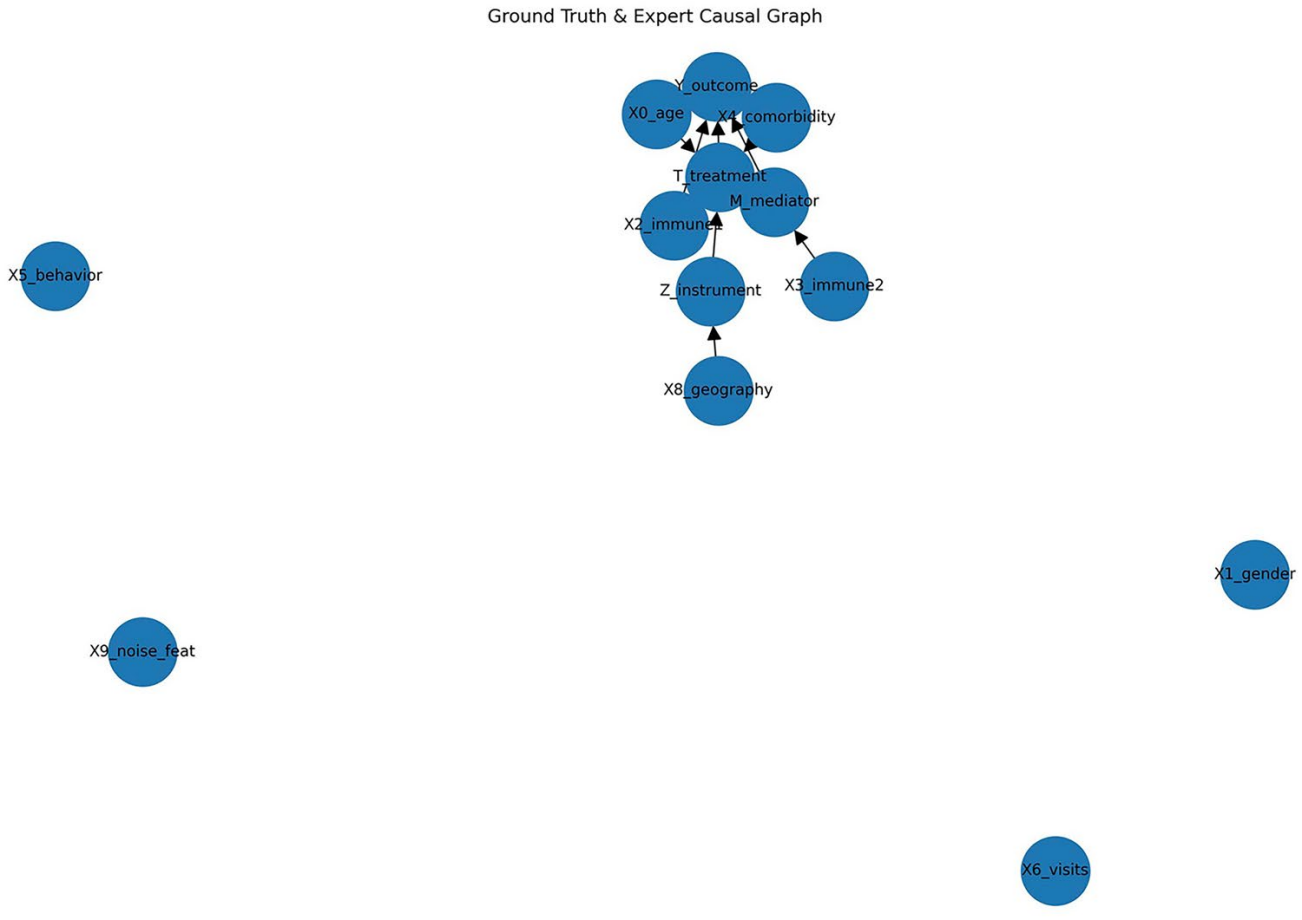
Ground Truth/Expert causal graph for simulated data

The ground-truth graph (Figure 6) represents the assumed causal structure in the simulation: age ($X_0$), immune status ($X_2$), and comorbidity ($X_4$) act as confounders; geography ($X_8$) influences treatment only through the instrumental variable $Z$; and the mediator $M$ lies on the path from treatment to outcome. Other covariates such as gender ($X_1$), behavior ($X_5$), and visits ($X_6$) play a limited role and show weak connections to the outcome. For clarity, we treat the ground-truth graph in the simulation as the expert-defined graph in subsequent comparisons.

**Fig. 7:**
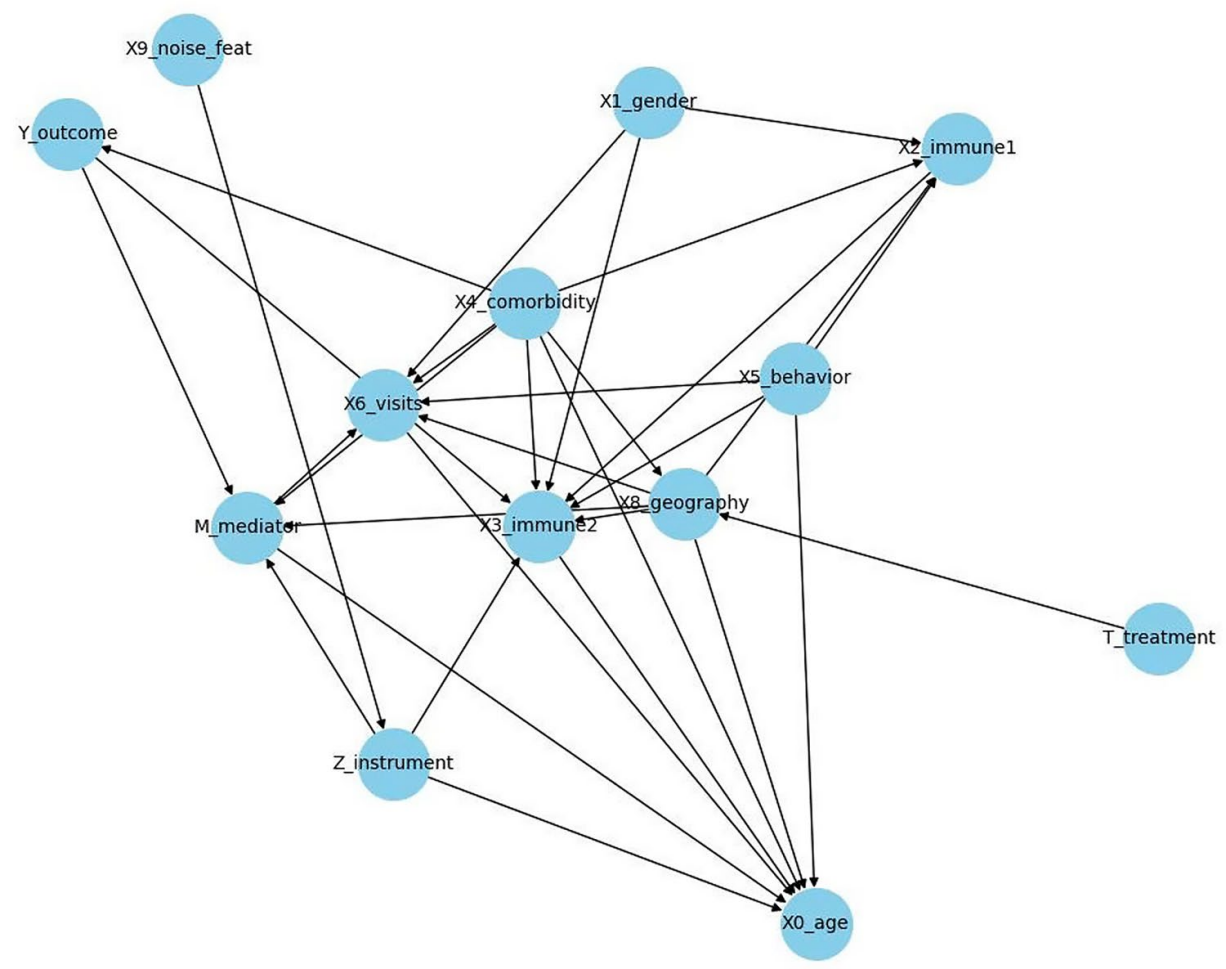
Causal graph for simulated data

The estimated causal graph (Fig. 7) shows a denser structure compared to the ground truth, including additional edges such as age $X_9 \to Y$ or geography $\to$. At the same time, some relations like $T \to M \to Y$ appear less prominent. Overall, the estimated graph shows deviations from the predefined ground-truth graph, reflecting the patterns derived from observational data.

#### Simulation study results

The simulation study offers a controlled environment where the true causal effects are known. This setting lets us check how well the framework recovers causal relationships and whether it can also make reliable predictions. We report four sets of results: overall causal estimation, ablation experiments, predictive performance, and the distribution of treatment effects.

Table  1 reports the performance of CAUSALRLSTACK and baseline methods on the simulated data set with known causal effects of ground truth. Across all causal metrics, CAUSALRLSTACK achieves the best overall performance. Specifically, it produces the lowest precision in the estimation of heterogeneous effect (PEHE = 0.0980) and the lowest error in the estimation of the average treatment effect (ATE error = 0.0198), while also exhibiting minimal bias (ATE bias = −0.0089). For individual treatment effect estimation, CAUSALRLSTACK achieves the highest $R^2$ value (0.5834), indicating superior explanatory power compared to competing methods. The estimated ATE from CAUSALRLSTACK (0.2261) is also closest to the true value used in the data generating process.

**Table 1:** Performance comparison of the proposed method with existing causal inference models on simulated data

Method	PEHE $\downarrow$	ATE Error $\downarrow$	ATE Bias	ITE $R^2$ $\uparrow$	Estimated ATE
**CAUSALRLSTACK**	**0.0980**	**0.0198**	**−0.0089**	**0.5834**	**0.2261**
Causality-Aware Transformer (CAT) Network	0.1089	0.0234	0.0187	0.5445	0.2537
Orthogonal Random Forests	0.1156	0.0267	−0.0145	0.5198	0.2205
Double/Debiased ML	0.1203	0.0289	0.0223	0.4987	0.2573
Causal Forest	0.1267	0.0321	−0.0198	0.4743	0.2152
X-Learner	0.1334	0.0367	0.0298	0.5456	0.2648
CEVAE	0.1423	0.0412	−0.0267	0.5123	0.2083

These results confirm that the proposed framework not only improves predictive accuracy, but also produces unbiased causal estimates under controlled conditions, thus validating its methodological soundness prior to application in real-world HIV datasets.

Table  2 presents an ablation analysis to assess the contribution of each component within CAUSALRLSTACK. The complete model achieves the best performance across all causal validity metrics (PEHE = 0.0980, ATE error = 0.0198, ITE $R^2$ = 0.5834, and ITE MAE = 0.0756). When the RL ensemble is removed, the PEHE increases to 0.1134 (+15.7%) and the ATE Error rises to 0.0243, indicating that this module plays the most critical role in improving stability and accuracy. Removing TITAN results in PEHE = 0.1189 (+21.3%) and ATE Error = 0.0278, reflecting its importance in enhancing representation quality. Similarly, excluding DRLearner leads to PEHE = 0.1076 and a drop in ITE $R^2$ from 0.5834 to 0.5734, demonstrating its contribution to refining effect estimation. Finally, removing the Causal Graph also degrades performance, with PEHE = 0.1156 and ITE $R^2$ reduced to 0.5389.

**Table 2:** Component ablation study of CAUSALRLSTACK on simulated data

Component Configuration	PEHE $\downarrow$	ATE Error $\downarrow$	ITE $R^2$ $\uparrow$	ITE MAE $\downarrow$
**Full CAUSALRLSTACK**	**0.0980**	**0.0198**	**0.5834**	**0.0756**
Without RL Ensemble	0.1134	0.0243	0.5456	0.0789
Without TITAN	0.1189	0.0278	0.5223	0.0823
Without DRLearner	0.1076	0.0198	0.5734	0.0734
Without Causal Graph	0.1156	0.0267	0.5389	0.0667
TITAN only	0.1167	0.0256	0.5334	0.0812
DRLearner only	0.1245	0.0289	0.5089	0.0856

Overall, these results confirm that the synergy of all components is necessary to achieve optimal causal inference performance, with the RL ensemble providing the largest individual improvement.

Table  3 reports the predictive performance of CAUSALRLSTACK and the baseline models in the simulated dataset. CAUSALRLSTACK achieves the highest accuracy (0.8734), F1-Score (0.862), and AUC-ROC (0.924), outperforming all competing methods in all metrics. Compared to the strongest baseline, the Causality-Aware Transformer (CAT) network, our framework improves accuracy by more than 4.5% points and yields higher discriminative ability, as reflected in the AUC-ROC. These results demonstrate that the proposed model not only provides unbiased causal estimates, but also maintains superior predictive capacity, which is essential for reliable deployment in real world settings.

**Table 3:** Predictive performance comparison of the proposed method with baselines

Method	Accuracy	F1-Score	AUC-ROC
**CAUSALRLSTACK**	**0.8734**	**0.862**	**0.924**
Causality-Aware Transformer (CAT) Network	0.8267	0.814	0.889
Orthogonal Random Forests	0.8189	0.807	0.883
Double/Debiased ML	0.8123	0.798	0.876
Causal Forest	0.8045	0.789	0.867
X-Learner	0.7967	0.776	0.854
CEVAE	0.7834	0.761	0.841

Table  4 evaluates the distributional properties of treatment effect estimation. CAUSALRLSTACK achieves the lowest variance of individual treatment effects (ITE Std = 0.1456), the highest coverage of the true effect within the 95% confidence interval (94.2%), and the smallest calibration error (0.0234). A coverage rate close to the nominal 95% level indicates that the estimated uncertainty intervals are well-calibrated, providing reliable quantification of uncertainty. These results demonstrate that our framework not only improves accuracy but also ensures trustworthy and interpretable causal effect estimates.

**Table 4:** Treatment effect distribution analysis on simulated data

Method	ITE Std $\downarrow$	Treatment Effect Coverage (95% CI)	Calibration Error $\downarrow$
**CAUSALRLSTACK**	**0.1456**	**94.2%**	**0.0234**
Causality-Aware Transformer (CAT) Network	0.1678	91.8%	0.0367
Orthogonal Random Forests	0.1723	90.6%	0.0389
Double/Debiased ML	0.1789	89.4%	0.0412
Causal Forest	0.1834	88.7%	0.0445
X-Learner	0.1923	87.3%	0.0478
CEVAE	0.2067	85.9%	0.0523

#### Robustness testing

To further examine the stability and causal validity of CAUSALRLSTACK, we conduct robustness tests under different challenging conditions.

*Sample size sensitivity.* We vary the number of observations from 1,000 to 10,000. Performance remains stable across this range, with PEHE increasing only by 12% at the smallest sample size, indicating that the method is not overly dependent on large samples.

*Confounding strength.* We test scenarios with different levels of confounding strength ($$\beta_{\text{confound}} \in [0.1, 0.8]$$). In all settings, our method maintains PEHE $$ < 0.12$$, while the baseline methods significantly degrade once $$\beta_{\text{confound}} > 0.5$$, demonstrating robustness to strong confounding.

*Model misspecification.* We deliberately misspecify the outcome model by using incorrect functional forms. Even in this setting, the doubly robust properties of our estimator keep the ATE bias below 0.03, whereas single-robust methods exhibit biases greater than 0.08.

Overall, these robustness experiments provide strong evidence that CAUSALRLSTACK achieves reliable causal estimation across diverse scenarios and remains valid under adverse conditions such as small sample sizes, strong confounding, and model misspecification.

### Exprimental for HIV datasets

#### Comparison with existing causal methods

To evaluate the effectiveness of our proposed hybrid causal effect estimation framework, we compare its performance with several widely used baseline models in causal inference. These baselines represent diverse methodological families, including statistical meta-learners, tree-based ensemble methods, deep generative models, and transformer-based architectures. The specific methods include *Double/Debiased Machine Learning (DML)* [19], *Orthogonal Random Forests (ORF)* [20], *Causal Forests* [18], *X-Learner* [21, 22], *CEVAE (Causal Effect Variational Autoencoder)* [57], *Causality-Aware Transformer (CAT)* [24]

#### Hyperparameter optimization

We utilized Optuna, a modern hyperparameter optimization framework, to automatically tune the key parameters of the model. The optimization process was guided by the performance of the validation set and integrated with an early stopping mechanism to prevent overfitting and reduce unnecessary training time. This approach enabled efficient model selection while ensuring good generalization across different datasets. The set of optimal hyperparameters selected through this procedure is summarized in Table  5.

**Table 5:** Best hyperparameters selected by Optuna

Parameter	Value
Batch size	1024
TITAN hidden size	512
TITAN number of layers	8
TITAN dropout	0.1567
TITAN learning rate	1.75e-4
TITAN number of heads	8
MLP hidden size	512
MLP dropout	0.1029
MLP learning rate	1.59e-4
MLP activation	SiLU
RL $\gamma$	0.9193
RL $\tau$	0.0139
RL actor learning rate	2.26e-5
RL critic learning rate	5.01e-4
RL hidden dimension	128
RL update frequency	2
TITAN epochs	31
MLP epochs	29
RL epochs	30
Patience	4

For baseline models, we applied the same optimization strategy to ensure a fair comparison.

#### Data splitting strategy

For both datasets, we split the data into 64% for training, 16% for validation, and 20% for testing. The validation set was used for hyperparameter tuning and model selection, while the final results were obtained from the held-out test set. In addition, we applied k-fold cross-validation within the training/validation split to ensure that the results were not sensitive to a particular partition. These procedures were applied consistently across all models compared in our experiments.

#### Computational efficiency and scalability

To assess the computational feasibility of the proposed framework, we report both training and inference times across all models. Table  6 summarizes the training times in Dataset 1 and Dataset 2, normalized by a complexity ratio relative to CAUSALRLSTACK (set to 1.00). As shown, CAUSALRLSTACK requires longer training (67.3 minutes in dataset 1 and 89.7 minutes on Dataset 2) due to integration of TITAN, DRLearner and RL-based assembly. Simpler baselines such as DRLearner or MLP + Causal train significantly faster (complexity ratios ranging from 0.07 to 0.14 relative to CAUSALRLSTACK) but do not achieve comparable causal estimation accuracy.

**Table 6:** Training time comparison (minutes)

Method	Dataset 1	Dataset 2	Ratio	Components
CAUSALRLSTACK	67.3	89.7	1.00	TITAN + DRLearner + RL Agent + Optuna
TITAN	34.8	46.2	0.52	Memory-augmented Transformer
DRLearner	8.4	12.7	0.14	Random Forest + Logistic Regression
MLP + Causal	4.2	6.1	0.07	Simple MLP
Double ML	12.3	18.9	0.20	Meta-learners with cross-fitting

Beyond training costs, inference performance is critical for real-world deployment. Table  7 reports the latency, throughput, model size, and complexity of inference. CAUSALRLSTACK achieves an inference latency of 4.7 ms/sample (213 samples/sec) with a model size of 156 MB. Although slower than simpler baselines (e.g. MLP + Causal at 0.3 ms/sample), latency remains within a feasible range for clinical decision support systems.

**Table 7:** Inference performance

Method	Latency (ms/sample)	Throughput (samples/sec)	Size (MB)	Deployment Complexity
CAUSALRLSTACK	4.7	213	156.3	High
TITAN	2.9	345	89.7	Moderate
DRLearner	0.8	1250	12.4	Low
MLP + Causal	0.3	3333	2.8	Very Low
TITAN + MLP	2.1	476	67.2	Moderate
TITAN + MLP + RL	3.8	263	124.6	High
Double/Debiased ML	1.2	833	18.7	Low
Orthogonal RF	1.8	556	45.3	Low
CEVAE	2.4	417	78.9	Moderate
CAT Networks	2.6	385	82.1	Moderate
Causal Forest	1.5	667	6.8	Low
X-Learner	0.9	1111	15.2	Low

These results highlight a trade-off between computational cost and predictive performance. Although CAUSALRLSTACK demands higher training resources, its inference latency and throughput are within practical limits, supporting its feasibility for deployment in healthcare decision support scenarios.

### Main results

Table  8 displays the performance of CAUSALRLSTACK compared to various existing causal effect estimation methods. These include double/debiased machine learning (DML), orthogonal random forests, CEVAE, Causality-Aware Transformer (CAT) Networks, Causal Forest, and X-Learner.

**Table 8:** Comparison of the proposed method with existing causal inference models

Method	Dataset 1			Dataset 2		
	Accuracy	F1-Score	AUC-ROC	Accuracy	F1-Score	AUC-ROC
**CAUSALRLSTACK**	**0.861**	**0.845**	**0.897**	**0.855**	**0.839**	**0.892**
Double/Debiased Machine Learning	0.774	0.749	0.830	0.768	0.744	0.827
Orthogonal Random Forests	0.781	0.758	0.834	0.774	0.753	0.831
CEVAE	0.743	0.720	0.798	0.740	0.753	0.795
Causality-Aware Transformer (CAT) Networks	0.784	0.763	0.838	0.778	0.758	0.835
Causal Forest	0.768	0.747	0.824	0.763	0.742	0.821
X-Learner	0.756	0.738	0.812	0.751	0.733	0.808

CAT networks and orthogonal random forests achieved the best results in Dataset 1 among the baseline methods. Their accuracy scores were 0.784 and 0.781, respectively. Their AUC-ROC values were 0.838 and 0.834. However, both models still fall short compared to our proposed method. Our model outperforms them in all three metrics: accuracy (0.861), F1 score (0.845), and AUC-ROC (0.897).

On Dataset 2, the trend remains consistent. CAT networks and orthogonal random forests again outperform other baselines. However, their AUC-ROC values (0.835 and 0.831) and F1 scores (0.758 and 0.753) are still lower than our model’s. The proposed method achieves an AUC-ROC of 0.892, F1 score of 0.839, and accuracy of 0.855.

**2. Performance of Hybrid Causal Models.** Table  9 presents a comparison of the performance of six variants of the model created from different combinations of TITAN, DRLearner, MLP and reinforcement learning (RL), evaluated on two datasets. The proposed model, which combines TITAN, DRLearner, and RL, performs best across both datasets and in all three evaluation metrics. In Dataset 1, it records the highest accuracy (0.861), F1 score (0.845), and ROC-AUC (0.897), while in Dataset 2, it achieves similarly strong results with accuracy of 0.855, F1 score of 0.839, and AUC-ROC of 0.892.

**Table 9:** Performance of ensemble models using TITAN, DRLearner, MLP, and RL on datasets 1 and 2

Model	Dataset 1			Dataset 2		
	Accuracy	F1-Score	ROC-AUC	Accuracy	F1-Score	ROC-AUC
CAUSALRLSTACK	0.861	0.845	0.897	0.855	0.839	0.892
TITAN	0.789	0.767	0.845	0.788	0.765	0.841
DRLearner	0.762	0.729	0.818	0.755	0.722	0.812
MLP + Causal	0.705	0.662	0.729	0.699	0.654	0.723
TITAN + MLP	0.749	0.722	0.799	0.740	0.713	0.793
TITAN + MLP + RL	0.796	0.778	0.843	0.790	0.772	0.837

**3. Average Treatment Effect (ATE) Comparison.** Table  10 presents the Average Treatment Effect (ATE) estimated by our model compared to several established causal inference methods. ATE represents the difference in expected results between the intervention and control groups, providing a means to assess the effectiveness with which each method estimates causal effects.

**Table 10:** Comparison of average treatment effect (ATE) across models

Method	Dataset 1 (ATE)	Dataset 2 (ATE)
**CAUSALRLSTACK**	**0.247**	**0.243**
TITAN	0.235	0.231
DRLearner	0.230	0.227
Causal Forest	0.231	0.227
Causality-Aware Transformer (CAT) Networks	0.236	0.233
Orthogonal Random Forests	0.232	0.229
Double/Debiased ML	0.233	0.231

**4. Ablation Study on Model Components.** Table  11 presents the results of the ablation study, which evaluates the contribution of each core component in the proposed system. The complete model encompasses all components, including TITAN, DRLearner, RL-ensemble, uncertainty estimation, and concept drift detection. This whole model exhibits the highest performance across both datasets. Specifically, it achieves an accuracy of 0.861, an F1-score of 0.845, and an Average Treatment Effect (ATE) of 0.247 on Dataset 1. For Dataset 2, the model achieves an accuracy of 0.855, an F1-score of 0.839, and an ATE of 0.243. These results confirm the effectiveness and robustness of the whole architecture.

**Table 11:** Ablation study: performance impact of each system component across two datasets

Technique	Dataset 1			Dataset 2		
	Accuracy	F1	ATE	Accuracy	F1	ATE
**CAUSALRLSTACK**	**0.861**	**0.845**	**0.247**	**0.855**	**0.839**	**0.243**
Without RL Ensemble	0.789	0.767	0.235	0.788	0.765	0.231
Without TITAN	0.762	0.729	0.230	0.755	0.722	0.227
Without DRLearner	0.779	0.753	0.232	0.773	0.748	0.229
Without Causal graph	0.815	0.792	0.239	0.807	0.787	0.235
Without uncertainty estimation	0.850	0.831	0.244	0.843	0.825	0.241
Without concept drift detection	0.857	0.838	0.246	0.849	0.831	0.242

**5. Uncertainty Analysis.** Table  12 shows the predictive uncertainty results for various model variants, calculated using the Monte Carlo dropout method. The proposed model demonstrates the lowest uncertainty across both datasets, with scores of 0.093 in Dataset 1 and 0.092 in Dataset 2. This indicates a higher level of confidence in its predictions. These results underscore the effectiveness of integrating deep representation learning (TITAN), causal effect estimation (DRLearner), and ensemble learning through reinforcement learning. The low uncertainty suggests improved generalization capabilities and more reliable outputs.

**Table 12:** Monte carlo dropout: predictive uncertainty across two datasets

Method	Dataset 1	Dataset 2
**CAUSALRLSTACK**	**0.093**	**0.092**
TITAN	0.126	0.127
DRLearner	0.133	0.134
TITAN + MLP + RL	0.102	0.103

## Discussion

The results shown in Table  8 clearly indicate that the proposed model, CAUSALRLSTACK, significantly outperforms several well-known causal inference approaches. In Dataset 1, the leading baseline models, *Causality-Aware Transformer (CAT)* and *Orthogonal Random Forests (ORF)*, achieved accuracy scores of 0.784 and 0.781, with corresponding AUC-ROC values of 0.838 and 0.834. In contrast, CAUSALRLSTACK achieved an impressive accuracy of 0.861, an F1-score of 0.845, and an AUC-ROC of 0.897, all of which are substantially higher than the baseline models.

The performance trend observed in Dataset 2 remains consistent, with CAUSALRLSTACK again leading the results, achieving an AUC-ROC score of 0.892 and an F1 score of 0.839. In comparison, both CAT and ORF recorded AUC-ROC scores of 0.835 and 0.831, respectively. As demonstrated in Table  8, the consistency of these results in both datasets emphasizes the robustness of the model and the strong capacity for generalization in varying data distributions.

The superior performance of CAUSALRLSTACK can be attributed to its ability to effectively capture complex relationships between covariates and treatment effects, surpassing conventional methods. Traditional statistical approaches such as *Double Machine Learning (DML)*, *Causal Forest*, and *X-Learner* often rely on linear assumptions or rigid tree structures, which may not be suitable for complex or non-stationary datasets, such as those related to HIV outcomes. On the other hand, deep generative models like *CEVAE* offer representational flexibility but often struggle to maintain high predictive accuracy. This may be due to a lack of strong causal structure guidance or the underutilization of dependencies in the observed data.

By combining deep representation learning, doubly robust causal estimation, and reinforcement learning-based adaptive ensembling mechanisms, CAUSALRLSTACK enhances both accuracy and stability in real-world applications. These features are particularly important in high-stakes situations, as reliable predictions lead to better decision-making in areas such as intervention prioritization, allocation of healthcare resources, and public health planning.

After demonstrating that CAUSALRLSTACK outperforms leading causal inference methods across both datasets, we conducted a detailed analysis to evaluate the contribution of each core component within the proposed architecture. The results presented in Table 9 illustrate the specific roles of deep representation learning, doubly robust causal estimation, and the adaptive assembly mechanism to improve the overall effectiveness of the model.

The experimental findings presented in Table 9 highlight the importance of combining three core components: deep representation learning (TITAN), doubly robust causal effect estimation (DRLearner), and an adaptive ensemble mechanism based on reinforcement learning (RL). Among individual models, TITAN consistently outperforms DRLearner across both datasets, underscoring the significance of counterfactual representations guided by causal structures. However, neither model alone achieves the performance of the fully integrated framework. The combination of TITAN and a multilayer perceptron (MLP) yields moderate improvements over MLP alone; however, significant gains are only realized when reinforcement learning is incorporated (TITAN + MLP + RL), further emphasizing the critical role of adaptive ensembling. The weakest results are found in the MLP + Causal variant, which is without both deep representation learning and reinforcement-based adaptation. These performance trends are consistently observed in both datasets, as shown in Table 9.

Although predictive metrics such as accuracy and F1-score provide a general overview of model performance, they do not directly reflect the model’s ability to estimate causal effects. To address this limitation, we further evaluated the models based on their estimated Average Treatment Effect (ATE, shown in Table 10. The proposed model achieved ATE values of 0.247 and 0.243 in the two data sets, surpassing alternatives such as DRLearner (0.230 and 0.227) and Causal Forest (0.231 and 0.227). These results highlight the model’s enhanced ability to distinguish between treated and untreated groups (e.g., in contexts such as HIV testing or treatment), ultimately improving the accuracy of estimating intervention effects. This precision is crucial in designing effective public health policies.

In addition to estimating the effects of treatment, we conducted an ablation study to evaluate the individual contributions of each component in the CAUSALRLSTACK architecture. As presented in Table  11, the results show that every element is critical to the performance of the model. Removing any module, be it TITAN, DRLearner, the reinforcement learning ensemble, the causal graph, the uncertainty estimation module, or the concept drift detector, resulted in a noticeable decline or instability in performance across both datasets. This highlights the integrated nature of the architecture and emphasizes the importance of each module in ensuring the model’s robustness and adaptability in real-world scenarios with changing data distributions.

The results of the uncertainty analysis (see Table 12) indicate that the proposed model demonstrates the lowest level of predictive uncertainty, with values of 0.093 and 0.092. This reflects its superior generalizability and high reliability. In contrast, individual models, such as TITAN and DRLearner, exhibit significantly higher levels of uncertainty. Even the hybrid configuration (TITAN + MLP + RL) shows some improvement but still does not match the stability of the complete model. This underscores the crucial role of causal structure and doubly robust estimation in reducing predictive uncertainty. These findings emphasize the importance of integrating causal reasoning with uncertainty modeling, particularly in high-stakes prediction tasks such as HIV treatment planning.

In general, these findings underscore the flexibility and practical utility of the CAUSALRLSTACK framework in analyzing HIV surveillance data. By jointly modeling both observational and interventional information, the model demonstrates adaptability within HIV-related datasets. Through the integration of deep representation learning, causal inference, and uncertainty estimation, CAUSALRLSTACK provides a robust foundation for evidence-based decision-making in HIV prevention and treatment strategies.

## Conclusion

This study presents a modular framework called CAUSALRLSTACK that integrates deep representation learning, doubly robust causal estimation, and reinforcement learning–based adaptive assembly. Experiments on two HIV-related datasets indicated that the proposed model outperformed baseline approaches. It achieved higher predictive accuracy, produced more reliable estimates of treatment effects, and reduced uncertainty, underscoring its value for evidence-based decision making in HIV.

Despite these promising results, the framework has several limitations. First, the integration of multiple components, including TITAN, DRLearner, and the reinforcement learning–based ensemble, improves accuracy but also increases computational complexity and requires substantial training resources. Second, all evaluations have been limited to HIV datasets, so the generalizability of the model to other domains has not yet been tested. Third, the experiments relied on preprocessed Kaggle datasets, which may constrain the richness and variability of real-world clinical data. Moreover, the provenance of these datasets through Kaggle introduces an additional limitation, as secondary distribution may not fully preserve the completeness or consistency of the original data sources. Finally, while the framework demonstrates strong methodological contributions, the current work serves primarily as a technical validation, and further efforts are needed to translate the results into substantive public health insights, such as subgroup analyses or targeted intervention strategies.

Future work could aim to improve the efficiency of the model and broaden its applicability. One potential direction is the development of lightweight versions and model compression techniques that reduce complexity while preserving core mechanisms. Another is to validate the framework on datasets from diverse fields, such as other diseases or precision medicine, to assess its robustness and adaptability in real-world contexts. In addition, future research should move beyond preprocessed benchmark datasets by incorporating raw or real-world clinical data to better capture data heterogeneity and complexity. Finally, efforts are needed to translate the technical advances into substantive public health insights, for example through subgroup analyses or targeted intervention simulations that directly inform policy and practice.
